# Jab1 promotes immune evasion and progression in acute myeloid leukemia models under oxidative stress

**DOI:** 10.1172/JCI183761

**Published:** 2025-08-05

**Authors:** Nan Zhang, Qian Wang, Guopeng Chen, Li Liu, Zhiying Wang, Linlu Ma, Yuxing Liang, Jinxian Wu, Xinqi Li, Xiaoyan Liu, Fuling Zhou

**Affiliations:** 1 Department of Hematology, Zhongnan Hospital of Wuhan University, Wuhan, China.; 2 Department of Hematology, The Second Affiliated Hospital of Chongqing Medical University, Chongqing, China.; 3 College of Chemistry and Molecular Sciences and; 4 Research Center for Lifespan Health, Wuhan University, Wuhan, China.

**Keywords:** Cell biology, Hematology, Cancer gene therapy, Leukemias, Signal transduction

## Abstract

Acute myeloid leukemia (AML) is the most common hematological malignancy. Leukemia stem cells exhibit high levels of oxidative stress, with ROS being the primary products of this stress, inducing the expression of c-JUN activation domain-binding protein 1 (Jab1). Previous studies have demonstrated that Jab1, as a transcriptional coactivator of c-JUN, promotes the malignant progression of AML under oxidative stress. However, its role in immune evasion is still under investigation. Here, we observed that knocking out Jab1 reduced the expression of immune checkpoints in vivo, effectively overcoming the immune evasion of AML. Interestingly, the deletion of Jab1 had no impact on the maturation of normal hematopoietic cells in mice. Mechanistically, Jab1 directly activated IGF2BP3 by driving the transcription factor c-JUN, consequently modulated the m^6^A modification of LILRB4 mRNA, and promoted immune evasion in AML. Finally, CSN5i-3 effectively disrupted the signaling pathway mediated by Jab1, thereby restoring cellular immune surveillance and halting the progression of AML. Thus, our results highlight the functional role of Jab1 in supporting AML survival and support the development of targeted therapeutic strategies.

## Abstract 2

Knocking out Jab1 reduces immune checkpoints in AML, overcoming immune evasion and halting disease progression, without affecting normal blood cell maturation in mice.

## Graphical abstract

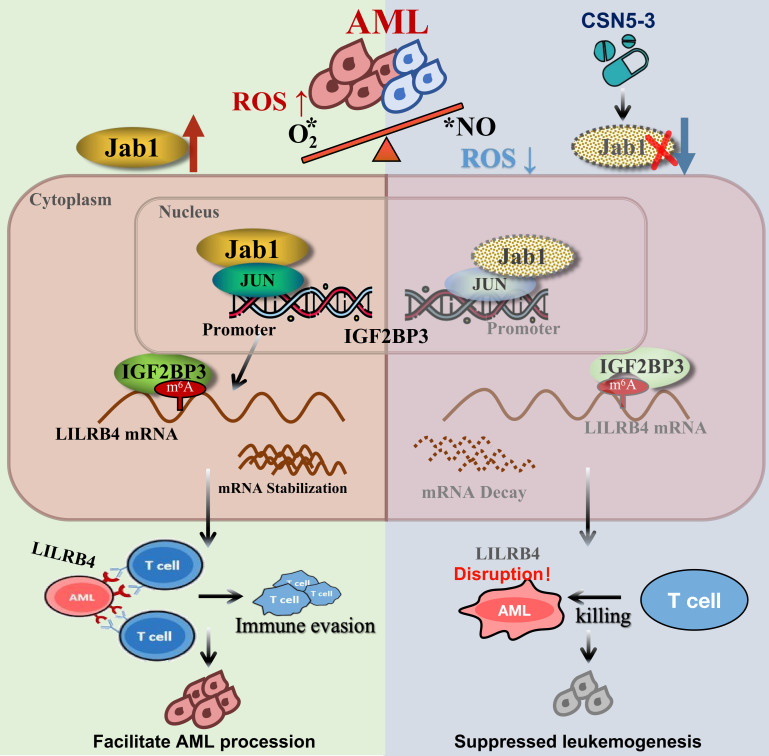

## Introduction

Acute myeloid leukemia (AML) is a hematologic malignancy that originates in the bone marrow and is characterized by the abnormal proliferation of primitive cells and an increase in immature granulocytes (1). ROS play a crucial role in balancing self-renewal of hematopoietic stem cells (HSCs) and myeloid differentiation (2). When this balance is disrupted, HSCs or their early progenitors are subjected to a cascade of genetic mutations and epigenetic abnormalities, leading to the development of leukemia (3). Our previous studies have shed light on some of the complex genetic mechanisms in AML, such as noncoding RNAs, RNA epigenetic modifications, and alternative splicing, providing insights into personalized medicine (4–6). However, further in-depth research is necessary to elucidate the pathogenic mechanisms underlying AML pathogenesis.

c-JUN activation domain-binding protein 1 (Jab1) was initially identified by our research group as a transcriptional coactivator of activator protein-1 (AP-1)/c-JUN and as the fifth component of the constitutive photomorphogenesis 9 (COP9) signalosome complex known as COPS5 (7, 8). Jab1 plays a crucial regulatory role in various signaling pathways by participating in gene transcription and protein degradation, thereby influencing multiple aspects of cellular function (9, 10). Structurally, Jab1 comprises a c-JUN binding domain, an Mpr1-Pad1-N-terminal domain containing a zinc metalloprotease motif (JAMM), a nuclear export signal domain, and a p27 binding domain at the C-terminus (8). In the regulation of apoptosis, Jab1 critically modulates p53 stability, with its deficiency leading to increased apoptosis, particularly in response to DNA damage (11, 12). Moreover, Jab1 is involved in regulating the expression of apoptosis-inducing proteins such as Fas ligand, BclGs, and E2F1 (13, 14). Our previous reports have highlighted the interaction between Jab1 and Trx under oxidative stress conditions, which promotes the progression and relapse of certain AML cases (15). Recent evidence suggests that Jab1 participates in AML progression by regulating immune evasion.

Here, we investigated the molecular mechanisms by which Jab1 contributes to AML progression and immune evasion. We highlight its roles in oxidative stress, RNA epigenetic modifications, and immune regulation. Leukemia stem cells showed markedly elevated ROS levels and increased Jab1 expression under ROS induction compared with normal progenitor cells. Knocking out Jab1 reduced immune checkpoint expression in vivo, overcoming AML immune evasion without affecting normal hematopoietic cell maturation. Jab1 activated IGF2BP3 by driving AP-1/c-JUN expression, regulating N6-methyladenosine (m^6^A) modification of leukocyte immunoglobulin-like receptor subfamily B member 4 (LILRB4) mRNA to promote AML progression. Our findings suggest that Jab1 may serve as a key regulatory factor in AML progression and is a potential therapeutic target.

## Results

### High oxidative stress drives Jab1 activation and AML development.

**Figure 1:**
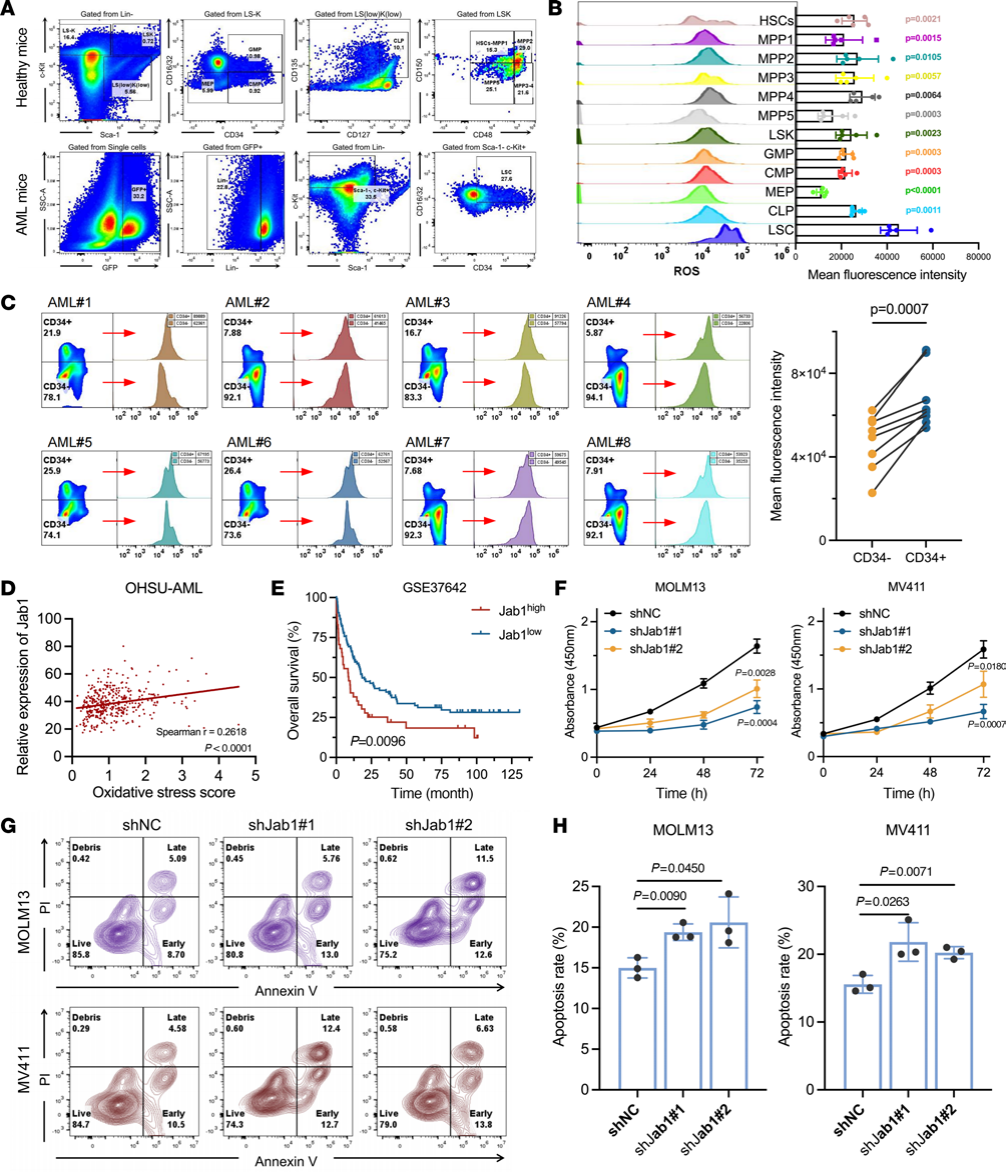
Jab1 is associated with oxidative stress and supports AML progression. (**A**) Flow cytometry strategy for the discrimination of hematopoietic stem/progenitor cells in normal murine bone marrow or AML stem cells. (**B**) ROS levels measured (*n* = 5 per group) by mean fluorescence intensity were significantly higher in LSCs than in other hematopoietic subsets (unpaired 2-tailed Student’s *t* test). (**C**) CD34^+^ cells from AML patients (*n* = 8) exhibit higher ROS levels than their CD34^–^ counterparts (*P* = 0.0007, by paired 2-tailed *t* test). (**D**) Jab1 expression positively correlates with oxidative stress scores in the OHSU-AML cohort (Spearman’s *r* = 0.2814, *P* < 0.0001). (**E**) High Jab1 expression is associated with poor overall survival in AML patients from GSE37642 (*P* = 0.0096) datasets (Kaplan-Meier analysis with log-rank test). (**F**) CCK-8 cell growth curve demonstrating that downregulation of Jab1 inhibits AML cell growth (72 hours absorbance compared using unpaired 2-tailed Student’s *t* test). (**G** and **H**) Flow cytometry analysis (*n* = 3 per group) of apoptosis and cell cycle in MOLM13 and MV411 cells upon Jab1 knockdown (unpaired 2-tailed Student’s *t* test). Values are presented as mean ±SEM.

Previous studies have linked AML progression and relapse to an imbalance in antioxidant capacity (15). To investigate the role of oxidative stress in HSC self-renewal and myeloid differentiation, we conducted FACS on normal hematopoietic stem/progenitor cells from mouse bone marrow and leukemia stem cells (LSCs) from mice transplanted with MLL-AF9 leukemia cells (Figure 1A). Quantitative analysis revealed that lower ROS levels are crucial for maintaining HSC self-renewal, a characteristic also observed in downstream MPP cells with enhanced stemness. Importantly, LSCs exhibited the highest ROS levels, significantly differing from other hematopoietic stem/progenitor cells (*P* < 0.01) (Figure 1B). These findings underscore the heightened oxidative stress in AML (15, 16). Although the surface markers of LSCs in patients with leukemia are still controversial, it is well known that LSCs are mainly rich in CD34^+^ cell populations in the bone marrow of patients (17–19). Consequently, we isolated CD34^+^ cells from primary AML patient samples and assessed their oxidative stress levels. Importantly, ROS levels in CD34^+^ cells from patients with AML were generally higher than those in CD34^–^ AML cells (Figure 1C), indicating the role of oxidative stress in LSC function and prompting our search for potential regulators of AML.

Subsequently, we constructed a risk scoring model for oxidative stress–related genes in AML (Supplemental Figure 1, A–G; supplemental material available online with this article; https://doi.org/10.1172/JCI183761DS1). We found that in AML patients with higher oxidative stress scores, Jab1 expression levels were significantly elevated (*P* < 0.0001) (Supplemental Figure 1H). Additionally, there was a significant positive correlation between Jab1 expression levels and oxidative stress scores (*P* = 0.0082) (Figure 1D). Analysis of the data suggested that Jab1 is widely expressed in AML, and high expression of Jab1 correlated with lower overall survival rates (Figure 1E and Supplemental Figure 2, A–E), consistent with previous studies (15). To further verify this, we performed multivariate Cox regression analysis. After adjusting for relevant clinical and molecular variables, Jab1 remained an independent predictor of poor prognosis (Supplemental Table 2). We analyzed Jab1 mRNA expression across AML cell lines using the Cancer Cell Line Encyclopedia database and confirmed by reverse transcription quantitative PCR (RT-qPCR) that MOLM13 and MV411 cells exhibited high Jab1 expression (Supplemental Figure 2, F and G). Knockdown of Jab1 in these cell lines significantly inhibited cell proliferation, increased apoptosis, and induced cell cycle arrest at the G_0_/G_1_ phase with a corresponding decrease in the S-G2-M phase population, whereas reexpression of Jab1 restored cell growth, reduced apoptosis, and reversed the cell cycle arrest phenotype (Figure 1, F–H, and Supplemental Figure 3, A–G). Collectively, these results indicate that Jab1 promotes AML cell growth in vitro by regulating proliferation.

Additionally, we established an oxidative stress model by intraperitoneally injecting d-galactose for 4–6 weeks at a daily dose of 200 mg/kg for 28 days. We observed significantly higher ROS levels in the d-galactose group compared with the control group (*P* = 0.0029) (Supplemental Figure 2, H and I). Under oxidative stress stimulation, the mice exhibited reduced body weight; decreased numbers of CD3^+^, CD4^+^, and CD8^+^ T cells in the spleen; and significantly increased expression of the T cell inhibitory receptors programmed cell death 1 (PD-1) and lymphocyte activation gene 3 (LAG-3) (Supplemental Figure 2, J–M). These findings warrant further investigation into the regulation of Jab1 during immune escape in AML under oxidative stress.

### Loss of Jab1 inhibits leukemogenesis and overcomes immune evasion in AML.

**Figure 2:**
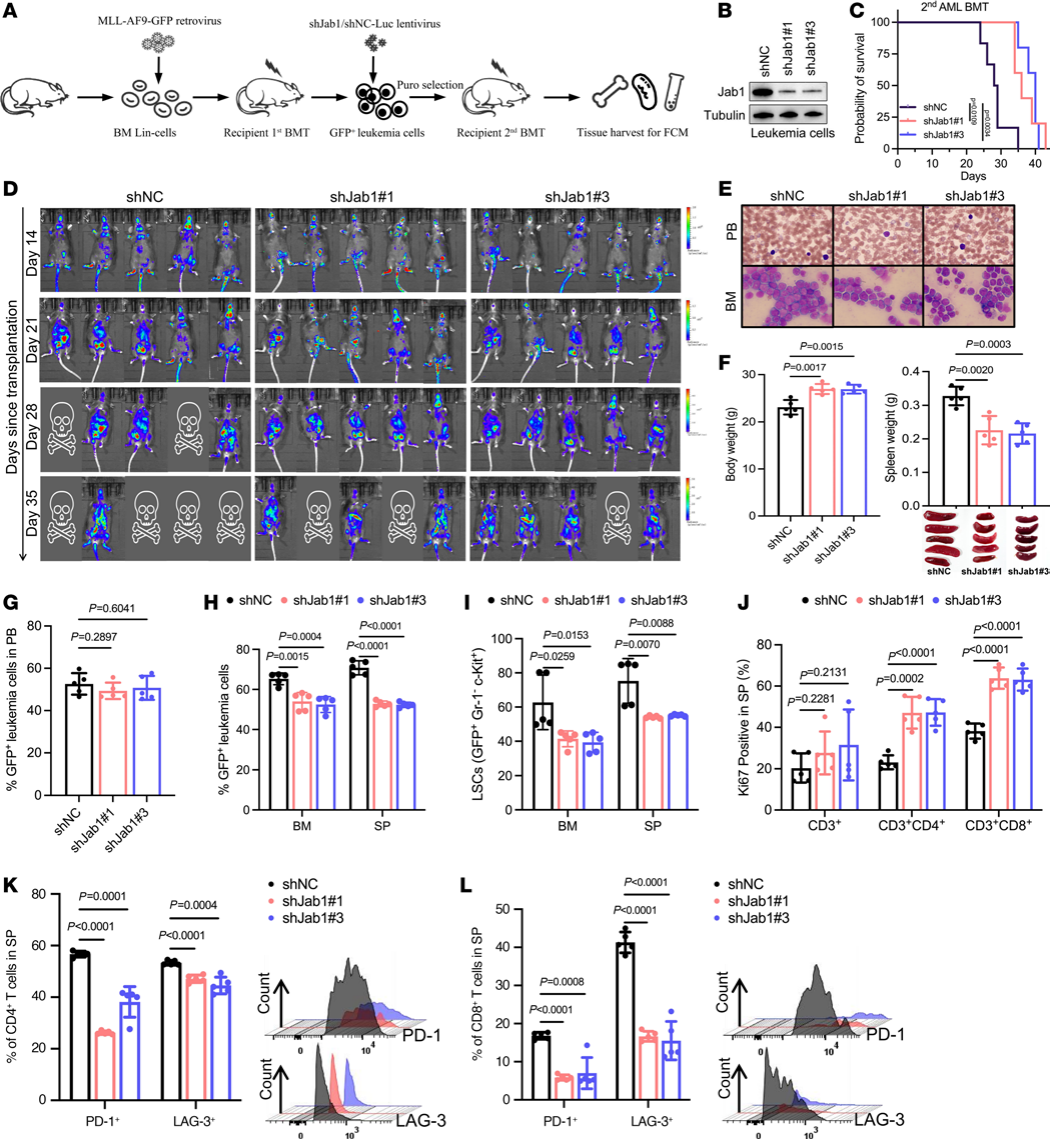
Jab1 facilitates immune evasion and sustains leukemia progression in MLL-AF9–driven AML. (**A**) Schematic representation of the experimental workflow for generating the MLL-AF9 AML mouse model and subsequent Jab1 knockdown using shRNA lentiviral vectors. (**B**) Western blot verifies effective Jab1 knockdown in leukemic cells. (**C**) Survival analysis shows that Jab1 silencing significantly prolongs survival in AML-bearing mice (Kaplan-Meier analysis with log-rank test). (**D**) In vivo bioluminescence imaging reveals reduced leukemic burden in Jab1-knockdown groups across multiple time points (days 14–35 after transplantation). (**E**) Wright-Giemsa staining shows decreased leukemic infiltration in peripheral blood (PB) and bone marrow (BM) of Jab1-deficient mice; original magnification, ×1,000. (**F**) Jab1 knockdown leads to increased body weight and reduced splenomegaly, indicating alleviated disease severity (*n* = 5 per group, by unpaired 2-tailed Student’s *t* test). (**G**–**I**) Flow cytometry shows that Jab1 knockdown leads to a significant reduction of GFP^+^ leukemic cells and LSCs (GFP^+^Gr-1^–^c-Kit^+^) in the bone marrow and spleen, while no significant difference was observed in the peripheral blood (*n* = 5 per group, by unpaired 2-tailed Student’s *t* test). (**J**) T cell proliferation, as measured by Ki-67 expression, is enhanced in CD4^+^ and CD8^+^ T cells upon Jab1 depletion in leukemic mice (*n* = 5 per group, by unpaired 2-tailed Student’s t test). (**K** and **L**) Jab1 silencing significantly reduces the proportion of PD-1^+^ and LAG-3^+^ exhausted CD4^+^ and CD8^+^ T cells in the spleen (*n* = 5 per group, by unpaired 2-tailed Student’s *t* test).

Next, we infected GFP^+^ second-generation MLL-AF9 mouse leukemia cells with lentivirus targeting Jab1 and performed secondary transplantation via tail vein injection to conduct in vivo and in vitro biological characterization (Figure 2, A and B). The results revealed that the knockdown of Jab1 significantly attenuated leukemia progression in mice, leading to an extended survival period (Figure 2, C and D). Wright-Giemsa staining of peripheral blood and bone marrow revealed a marked reduction in blast cell proportions in the Jab1-knockdown group (Figure 2E), along with alleviated splenomegaly and weight gain (Figure 2F). However, Jab1 knockdown did not significantly alter the percentage of GFP^+^ leukemia cells in peripheral blood (Figure 2G), but it notably reduced the infiltration of GFP^+^ leukemia cells and GFP^+^Gr-1^+^c-Kit^+^ LSCs in the bone marrow and spleen (Figure 2, H and I).

In investigating the role of Jab1 in immune evasion in leukemic mice, we assessed T cell proliferation, inhibitory receptor expression, T cell subset distribution, and subset cell proliferation (20). Knockdown of Jab1 in MLL-AF9–induced AML mice led to increased Ki67 positivity of spleen CD3^+^CD4^+^ and CD3^+^CD8^+^ T cells (Figure 2J). Expression of inhibitory receptors PD-1 and LAG-3 in spleen CD3^+^CD4^+^ and CD3^+^CD8^+^ T cells decreased markedly following Jab1 knockdown (Figure 2, K and L). Analysis of T cell subsets, categorized by CD44 and CD62L expression, revealed a rise in spleen CD3^+^CD4^+^ or CD3^+^CD8^+^ naive T (T_N_) cells in the Jab1-knockdown group, with a slight reduction in spleen CD3^+^CD4^+^ or CD3^+^CD8^+^ effector memory T (T_EM_) cells and a decrease in spleen CD3^+^CD8^+^ terminally differentiated effector memory T (T_EMRA_) cells (Supplemental Figure 4A). Jab1 knockdown enhanced Ki67 expression in CD3^+^CD4^+^ and CD3^+^CD8^+^ T cells in the spleen, indicating increased T cell subset activation (Supplemental Figure 4, B–D).

To directly distinguish whether this immune-modulatory effect was cell intrinsic or immune mediated, we further employed Rag1^–/–^ mice lacking mature T and B cells. Jab1 knockdown prolonged survival in both immunocompetent and Rag1^−/−^ recipients, though to a lesser extent in the latter (42 vs. 36 days), suggesting partial contribution from host immunity (Supplemental Figure 5, A–C). We used an immunocompetent C1498-derived AML model to validate Jab1’s role in disease progression. Jab1 downregulation reduced leukemic burden and delayed disease onset, accompanied by immune activation and increased T_N_ cell proportions in both spleen and bone marrow (Supplemental Figure 6, A–J). Overall, Jab1 downregulation significantly altered the immune microenvironment in mice, relieving immune suppression in AML and boosting T_N_ cell proportions in the spleen and bone marrow, along with enhancing proliferation in various immune cell subpopulations.

### Jab1 is dispensable for normal hematopoiesis and multilineage reconstitution.

**Figure 3:**
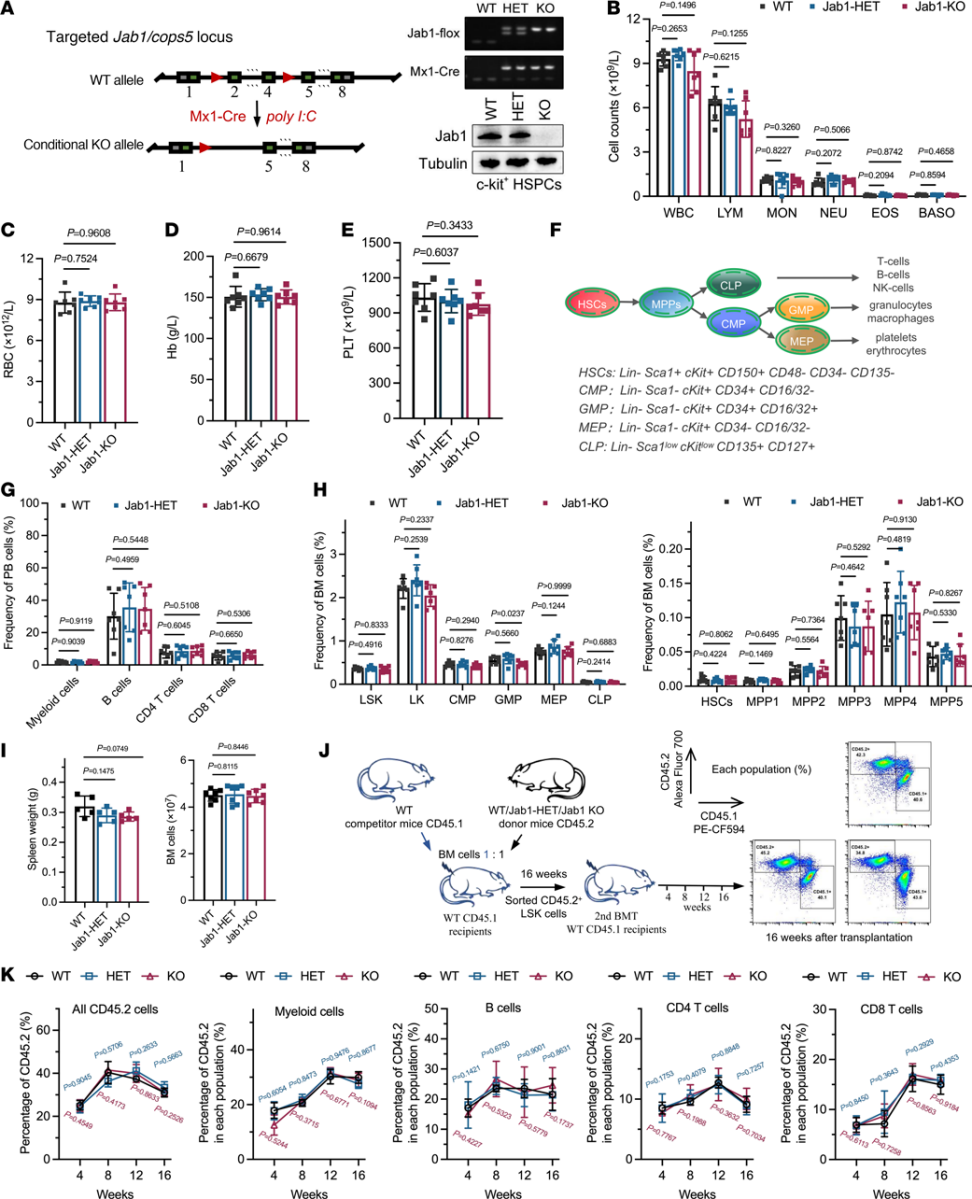
Jab1 is dispensable for normal hematopoiesis and multilineage reconstitution. (**A**) Generation of conditional Jab1-knockout mice (Mx1-Cre Jab1^fl/fl^) using poly(I:C)-induced recombination; successful deletion in c-Kit^+^ HSPCs was verified by Western blot. (**B**–**E**) Peripheral blood analysis shows no significant differences in leukocyte subtypes (WBC, lymphocyte [LYM], monocyte [MON], neutrophil [NEU], eosinophil [EOS], and basophil [BASO]), RBC count, hemoglobin (Hb), or platelet (PLT) levels among WT, Jab1-HET, and Jab1-KO mice (*n* ≥ 6 per group, by unpaired 2-tailed Student’s *t* test). (**F**) Schematic diagram of classical hematopoietic lineage differentiation. (**G**) Flow cytometry of peripheral blood reveals comparable proportions of myeloid cells, B cells, CD4^+^ T cells, and CD8^+^ T cells across all genotypes (*n* ≥ 6 per group, by unpaired 2-tailed Student’s *t* test). (**H**) Quantification of bone marrow progenitor and stem cell populations (LSK, LK, CMP, GMP, MEP, CLP, HSCs, and MPP1–MPP5) shows no significant disruptions in Jab1-deficient mice (*n* ≥ 6 per group, by unpaired 2-tailed Student’s *t* test). (**I**) Total bone marrow cell counts and spleen weights were not affected by Jab1 deletion, further supporting preserved hematopoiesis (*n* ≥ 6 per group, by unpaired 2-tailed Student’s *t* test). (**J** and **K**) Secondary competitive bone marrow transplantation assays demonstrate that Jab1-KO HSCs maintain long-term reconstitution capacity, with comparable CD45.2^+^ donor-derived contributions to all major blood lineages (myeloid, B, CD4^+^, and CD8^+^ T cells) up to 16 weeks after transplantation (primary transplantation results are shown in Supplemental Figure 9, C–I) (*n* ≥ 6 per group, by unpaired 2-tailed Student’s *t* test).

We investigated the impact of Jab1 loss on the hematopoietic system. Using CRISPR/Cas9 technology, we inserted 2 FloxP components into the mouse genome between exons 1 and 2, as well as exons 4 and 5 of the Jab1 gene. These FloxP sequences enabled specific recognition and cleavage of this region by the Cre protein. Through exogenous administration of polyinosinic:polycytidylic acid [poly(I:C)], we induced the expression of Cre enzyme, leading to complete deletion of the Jab1 gene (Figure 3A). At 4 weeks after the last poly(I:C) injection, both Jab1-HET and Jab1-KO mice exhibited comparable blood counts and frequencies of different blood cell types in peripheral blood as WT mice (Figure 3, B–E). Furthermore, there were no significant changes in the proportions of myeloid cells (Mac1^+^, Gr-1^+^), B cells (CD3^–^, B220^+^), CD4^+^ T cells (CD3^+^, CD4^+^), and CD8^+^ T cells (CD3^+^, CD8^+^) in peripheral blood (Figure 3G and Supplemental Figure 7, A and B). To assess the potential impact of Mx1-Cre activity on major organs, we specifically examined the kidneys, lungs, spleen, liver, and heart. Histopathological examination revealed no apparent defects or abnormalities in these organs (Supplemental Figure 7C), with no significant alterations in spleen size (Figure 3I and Supplemental Figure 7D). These findings suggest that Jab1 deficiency does not affect the number or proportion of mature cells in the normal hematopoietic system under steady-state conditions within the time frame examined.

HSCs are capable of self-renewal and multipotent differentiation, and there is a certain regularity in the developmental process of blood cells from the primitive to mature stages (Figure 3F). The total number of bone marrow cells was generally similar among WT, Jab1-HET, and Jab1-KO mice, ranging from 4 × 10^7^ to 5 × 10^7^ (Figure 3I). In mouse bone marrow, the percentages and total numbers of various cell populations, including LSK, LK, CMP, MEP, CLP, HSC, and MPP1-5, were similar to those in WT mice, except for GMP (Figure 3H, Supplemental Figure 8, A–C, and Supplemental Figure 9, A and B). GMP primarily differentiates into granulocytes and monocytes, and previous data indicate that Jab1 deficiency has no significant effect on MON, NEU, EOS, or BASO, which explain the inhibitory effect of Jab1 loss on less-differentiated myeloid progenitor cells. These findings suggest that Jab1 deficiency may delay AML cell progression to some extent, without interfering with normal bone marrow cell maturation.

To assess the impact of Jab1 on HSC self-renewal capacity, we conducted a competitive repopulation assay (Supplemental Figure 9C). The proportions of total cells (CD45.2^+^), myeloid cells, B cells, CD4^+^ T cells, and CD8^+^ T cells from WT, Jab1-HET, and Jab1-KO donors in peripheral blood were comparable 4–16 weeks after bone marrow transplantation (Supplemental Figure 9, D–H). Furthermore, at the 16-week time point, no notable differences were observed in the percentages of stem and progenitor cells derived from donors (Supplemental Figure 9I). To further evaluate long-term hematopoietic function under stress conditions, we isolated donor-derived LSK cells 16 weeks after transplantation and conducted secondary transplantation into lethally irradiated recipients. Jab1-deficient HSCs sustained multilineage engraftment over 16 weeks with reconstitution efficiency comparable with controls (Figure 3, J and K). In contrast, in the MLL-AF9–induced AML model, Jab1 deletion markedly delayed disease progression and prolonged survival in both primary and secondary transplant recipients (Supplemental Figure 9, J and K), highlighting Jab1’s role in leukemogenesis rather than in normal hematopoiesis.

### Jab1 affects LILRB4 mRNA stability and contributes to immune escape in AML.

**Figure 4:**
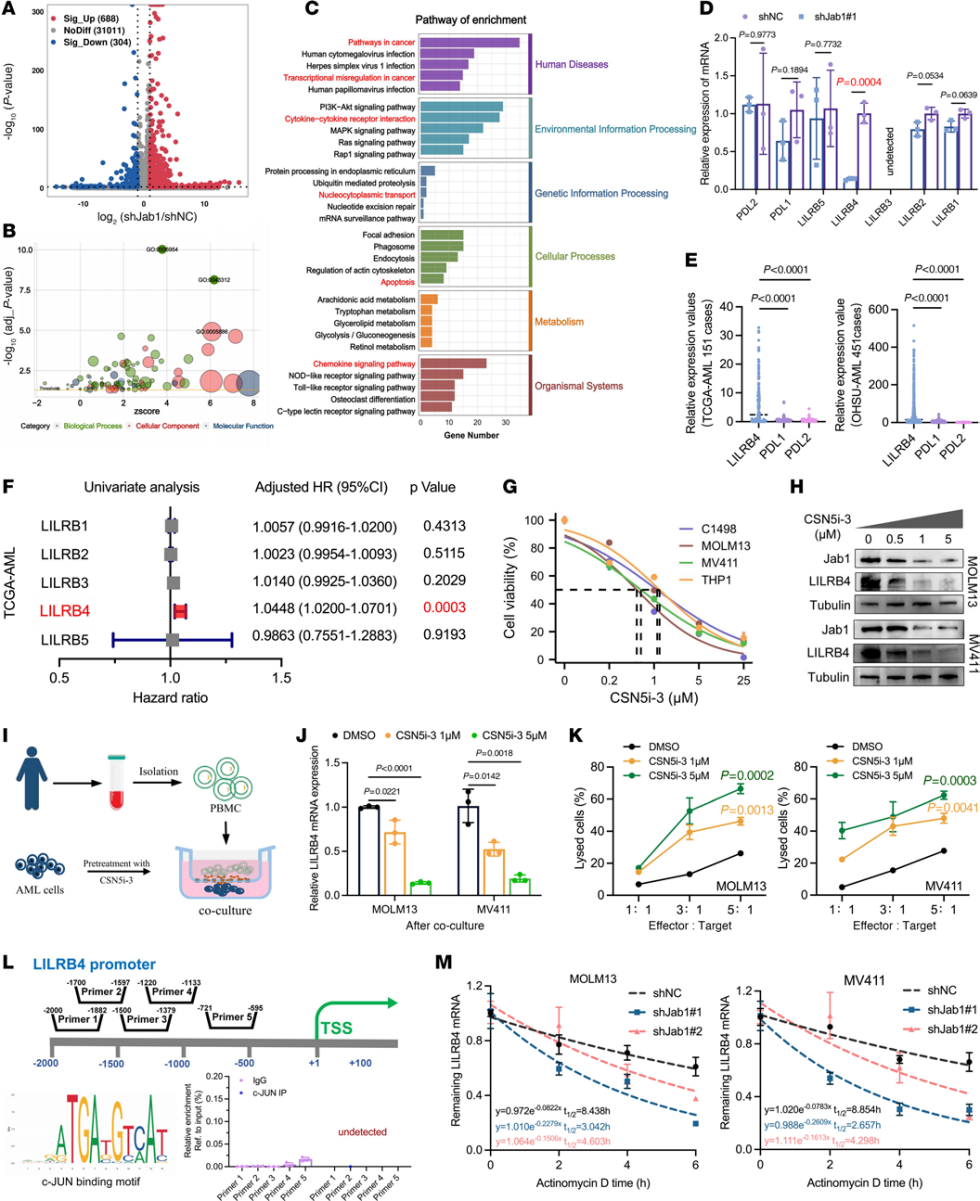
Jab1 stabilizes LILRB4 mRNA and promotes immune evasion in AML. (**A**–**C**) Transcriptomic analysis in MOLM13 cells with Jab1 knockdown reveals significant changes in gene expression, enriched in immune-related Gene Ontology terms and KEGG (Kyoto Encyclopedia of Genes and Genomes) pathways including cytokine signaling and leukocyte activation. (**D**) RT-qPCR validation confirms downregulation of LILRB4 upon Jab1 depletion, while PD-L1, PD-L2, and other LILRB family members remain largely unchanged (*n* = 3 per group, by unpaired 2-tailed Student’s *t* test). (**E**) Relative expression levels of PDL1, PDL2, and LILRB4 in TCGA-AML (*n* = 151) and OHSU-AML (*n* = 451) datasets (by unpaired 2-tailed Student’s *t* test). (**F**) Univariate Cox regression shows high LILRB4 expression is associated with poorer overall survival in AML (*P* = 0.0003). (**G** and **H**) CSN5i-3, a Jab1 inhibitor, reduces cell viability in multiple AML cell lines and downregulates LILRB4 protein expression in a dose-dependent manner. (**I**–**K**) Coculture of CSN5i-3–pretreated AML cells with PBMCs enhances LILRB4 suppression and promotes T cell–mediated leukemia lysis across multiple effector/target ratios (*n* = 3 per group, by unpaired 2-tailed Student’s *t* test). (**L**) ChIP-qPCR analysis of c-JUN transcription factor binding motifs and LILRB4 promoter sequence to detect enrichment of LILRB4 in c-JUN complexes. TSS, transcription start site. (**M**) Assessment of LILRB4 mRNA stability and half-life in Jab1 knockdown MOLM13 and MV411 cells treated with 5 μg/mL actinomycin D at 0, 2, 4, and 6 hours.

From our findings, we observed that downregulating Jab1 expression in AML cells alleviated the immunosuppressive microenvironment. Surprisingly, the deletion of Jab1 in hematopoietic cells did not affect immune cell maturation and development. Thus, we hypothesized that Jab1 likely does not directly influence immune cell function but instead regulates specific immunosuppressive targets in AML cells (21). High-throughput sequencing revealed that 688 genes were significantly upregulated and 304 genes were significantly downregulated in the Jab1-knockdown group (Figure 4A). Pathway analysis indicated that differentially expressed mRNAs were predominantly enriched in pathways associated with cancer, transcriptional misregulation in cancer, cytokine–cytokine receptor interaction, nucleocytoplasmic transport, apoptosis, and chemokine signaling (Figure 4, B and C). Initially, we validated the commonly observed immune checkpoint targets programmed cell death ligands 1 and 2 (PD-L1 and PD-L2) in tumor cells; however, no significant differences were observed (Figure 4D). Given these results, we redirected our research focus to the LILRB family, an emerging set of immune checkpoint targets in AML (22). Our findings demonstrated that LILRB4 is regulated by Jab1 (Figure 4D), a target extensively associated with immune evasion in AML (7, 22, 23). Additionally, the expression of PD-L1 and PD-L2 in AML was extremely low, while LILRB4 expression was significantly higher (Figure 4E). These findings partially elucidate the limited efficacy observed in clinical trials of PD1/PD-L1/PD-L2 immunotherapy strategies targeting AML. Further analysis revealed a correlation between high LILRB4 expression and poorer overall survival (Figure 4F and Supplemental Figure 11A), supported by survival data from multiple datasets (Supplemental Figure 12, A–E). Jab1 knockdown effectively reduced LILRB4 positivity in THP1, MOLM13, and MV411 cells, without significantly affecting the expression of PD-L1 and PD-L2 (Supplemental Figure 10, A–F). Notably, Jab1 deletion did not affect LILRB4 expression in normal c-Kit^+^ hematopoietic stem and progenitor cells (HSPCs) (Supplemental Figure 7, E and F). Similar experiments conducted on previously established MLL-AF9 mice showed that Jab1 regulates LILRB4 expression in GFP^+^ leukemia cells and GFP^+^Gr-1^–^c-Kit^+^ leukemia cells, with Jab1 knockdown resulting in decreased LILRB4 expression (Supplemental Figure 11B).

Jab1 is pivotal in AML development but not in normal hematopoiesis, making it a promising therapeutic target. CSN5i-3 selectively inhibits Jab1 (Supplemental Figure 12F), with IC_50_ values ranging from 0.5364 to 1.118 μM for MOLM13, THP1, MV411, and C1498 cells (Figure 4G). This emphasizes the effectiveness of CSN5i-3 in reducing AML cell viability, suggesting its therapeutic promise for AML (24). To further evaluate the therapeutic window, we assessed the drug’s cytotoxicity in normal human CD34^+^ HSPCs. The IC_50_ values in HSPCs ranged from approximately 11 to 26 μM, which is over 10-fold higher than those observed in AML cells (Supplemental Figure 12G). This indicates a favorable selectivity index and suggests that CSN5i-3 preferentially targets leukemic cells while sparing normal hematopoietic progenitors. Additionally, increasing concentrations of CSN5i-3 led to more pronounced degradation of Jab1 protein (Figure 4H), accompanied by elevated apoptosis rates, altered cell cycle distribution, and enhanced differentiation (Supplemental Figure 12, H–J). Notably, treatment with either CSN5i-3 or the AP-1 inhibitor T-5224 markedly reduced LILRB4 protein levels, whereas the neddylation inhibitor MLN4924 had no such effect (Supplemental Figure 12K), indicating that Jab1 regulates LILRB4 expression primarily through its role as a transcriptional coactivator rather than via its deneddylase activity. These findings highlight the concentration-dependent growth inhibition of AML cells induced by the drug. In vitro coculture experiments subsequently showed that CSN5i-3 significantly reduced LILRB4 mRNA expression in AML cells, enhancing immune cell cytotoxicity against them (Figure 4, I–K). Additionally, CSN5i-3 treatment led to a notable decrease in PD-1 and Tim-3 expression in T cells (Supplemental Figure 11, C and D), indicating its potential to mitigate T cell immunosuppression, thus enhancing its anti-AML efficacy.

These findings encouraged us to further explore the specific regulatory mechanisms through which Jab1 influences LILRB4. Given that Jab1 acts as an AP1/c-JUN transcriptional coactivator, we initially hypothesized that Jab1 affects the expression of LILRB4 through transcriptional regulation (7, 8). We identified 5 potential binding sites of c-JUN transcription factors within the promoter sequence of LILRB4 (NC_000019.10:54660985-54663085). However, ChIP experiments aimed at extracting c-JUN complexes from AML cells did not demonstrate significant enrichment of LILRB4 (Figure 4L). Surprisingly, downregulating Jab1 significantly accelerated the degradation of LILRB4 mRNA (Figure 4M). These findings suggest that Jab1 might influence LILRB4 expression through indirect mechanisms.

### Jab1 regulation of IGF2BP3 transcription influences the m^6^A modification of LILRB4.

**Figure 5:**
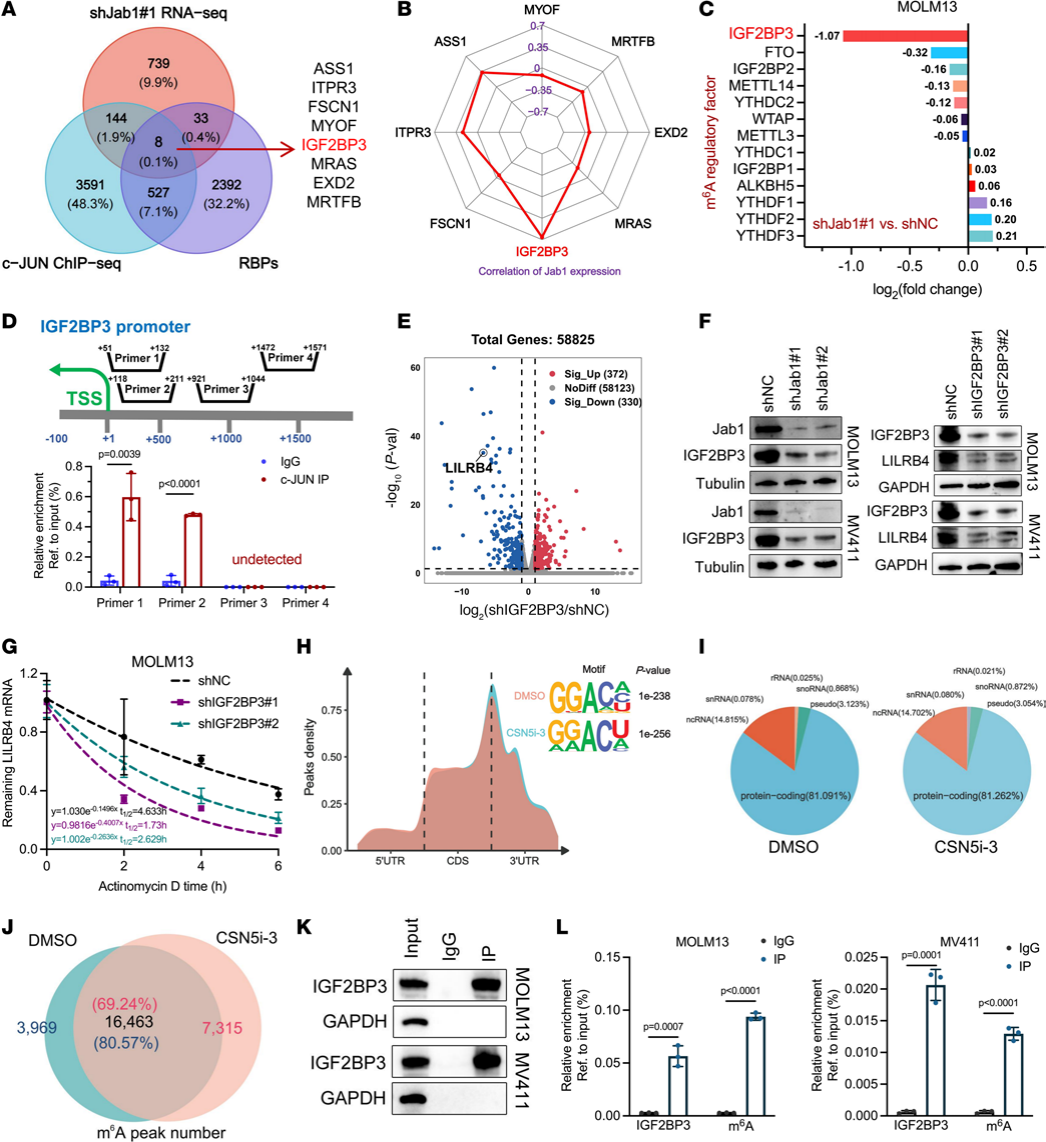
Jab1 transcriptionally regulates IGF2BP3 to control m^6^A modification and stability of LILRB4 mRNA. (**A**) Venn diagram showing the overlap between genes identified from Jab1 knockdown RNA-Seq, c-JUN ChIP-Seq, and known RBPs, highlighting IGF2BP3 as a candidate. (**B**) Radar plot depicting correlation of candidate genes with Jab1 expression in AML samples from the GSE12662 dataset. (**C**) Jab1 knockdown alters the expression of several m^6^A regulatory factors in MOLM13 cells, with IGF2BP3 showing the most notable reduction. (**D**) ChIP-qPCR reveals c-JUN binding enrichment at the IGF2BP3 promoter, suggesting transcriptional regulation of IGF2BP3 by Jab1 through c-JUN. TSS, transcription start site. (**E**) RNA-Seq analysis shows that LILRB4 is one of the most significantly downregulated genes following IGF2BP3 knockdown in MOLM13 cells. (**F**) Western blot validation confirms that Jab1 knockdown reduces IGF2BP3 protein levels and that IGF2BP3 silencing leads to reduced LILRB4 expression. (**G**) Actinomycin D chase assay shows that IGF2BP3 knockdown accelerates LILRB4 mRNA decay, indicating its role in mRNA stabilization. (**H**) MeRIP-Seq metagene analysis shows m^6^A peak distribution across transcripts and identifies the conserved GGACU motif in MOLM13 cells treated with CSN5i-3. CDS, coding sequence. (**I** and **J**) Pie and Venn diagrams display the proportion and overlap of m^6^A-modified RNA species in MOLM13 cells following CSN5i-3 treatment. (**K**) Western blot validation of specific bands in cell lysates and IGF2BP3-RIP complexes, evaluating the effectiveness of RIP products. (**L**) RIP-qPCR analysis quantifies enrichment of LILRB4 in IGF2BP3 and m^6^A immunoprecipitates, confirming IGF2BP3 binding to m^6^A-modified LILRB4 mRNA.

Processes influencing mRNA stability are typically regulated by RNA binding proteins (RBPs) (25–27). Therefore, we retrieved 2,960 RBPs from EuRBPDB (28), analyzed RNA-Seq data after Jab1 knockdown to identify differentially expressed RBPs, and compared them with c-JUN ChIP-Seq data to identify 8 potential regulatory factors (Figure 5A). Further analysis showed a strong correlation between IGF2BP3 and Jab1 expression (Figure 5B). Literature review indicated that IGF2BP3, functioning as an m^6^A recognition protein, protects m^6^A-modified RNA from degradation by binding to crucial regions of the target mRNA (such as MYC, CEBPA, BCL2, etc.), thereby promoting RNA stability and oncogenic effects (17, 29, 30). Notably, among all m^6^A regulatory factors affected by Jab1 knockdown, the most significant decrease was observed in IGF2BP3 (Figure 5C). The results indicate enrichment of c-JUN at the +51 to +132 and +118 to +211 sites of IGF2BP3. This suggests that Jab1 may regulate the expression of IGF2BP3 by recruiting the transcription factor c-JUN (Figure 5D). In a retrospective analysis of prior RNA-Seq data after IGF2BP3 knockdown (5), we observed that LILRB4 expression was similarly affected (*P* = 2.78 × 10^–45^, Log_2_FoldChange = –6.76) (Figure 5E). Furthermore, validation showed that Jab1 knockdown decreased IGF2BP3 protein expression, and IGF2BP3 knockdown reduced LILRB4 protein expression (Figure 5F). These results highlight the interaction between Jab1 and IGF2BP3 and their role in regulating LILRB4 expression.

High expression of IGF2BP3 in AML was associated with poorer clinical outcomes (Supplemental Figure 13, A–D). Knockdown of IGF2BP3 accelerated the degradation of LILRB4 mRNA, indicating its role in maintaining LILRB4 stability (Figure 5G and Supplemental Figure 13E). Additionally, analysis of methylated RNA immunoprecipitation sequencing (MeRIP-Seq) and RIP-Seq data from GSE76414 and GSE60213 suggested that IGF2BP3 is enriched in m^6^A-modified LILRB4 mRNA (16, 31). To identify transcripts modulated by the Jab1 inhibitor CSN5i-3, we conducted MeRIP-Seq assays on MOLM3 cells treated with 1 μM CSN5i-3. Consistent with prior m^6^A-seq findings, we observed abundant m^6^A peaks within mRNA open reading frames, around stop codons, and in 3′-UTR regions, displaying the classic DRACH (D, A/G/U; R, A or G; H, A/C/U) motif (Figure 5H). Moreover, over 81% of m^6^A-tagged transcripts encoded proteins (Figure 5I). Following CSN5i-3 treatment, the number of m^6^A-tagged transcripts notably increased, with 16,463 transcripts detected both before and after treatment, constituting 69.24% of the transcripts after CSN5i-3 treatment (Figure 5J). Integrative genomic observations indicated that CSN5i-3 reduced the peak enrichment of highly abundant m^6^A in the LILRB4 transcripts (Supplemental Figure 13F). This suggests that CSN5i-3 may influence the modification levels of LILRB4 in leukemia cells, thereby affecting its expression. Previous studies have indicated that IGF2BP3, acting as an RBP and m^6^A reader, can directly bind to mRNA and promote RNA stability, exerting oncogenic effects (5, 32). Validation through RIP experiments targeting the IGF2BP3/m^6^A antibody revealed increased enrichment of LILRB4 mRNA (Figure 5, K and L). Additionally, key regulators of apoptosis and the cell cycle — BCL2, XIAP, and E2F1 — were also found to be modulated by the Jab1/IGF2BP3 axis (Supplemental Figure 13, G and H), suggesting that this pathway may stabilize oncogenic m^6^A-modified mRNAs beyond LILRB4.

### Overexpression of IGF2BP3 rescues the growth inhibition of AML caused by Jab1 deficiency.

To further validate the regulatory mechanism of the Jab1/IGF2BP3/LILRB4 signaling axis in AML progression and immune evasion, rescue experiments were conducted. Remarkably, overexpressing IGF2BP3 successfully countered the decrease in LILRB4 induced by Jab1 deficiency (Supplemental Figure 14, A and D). Cell proliferation assays revealed that IGF2BP3 overexpression promoted AML cell proliferation, suggesting its pivotal role in AML growth processes. Additionally, IGF2BP3 overexpression mitigated the growth inhibition of AML cells caused by Jab1 deficiency (Supplemental Figure 14, B and E). Flow cytometric apoptosis analysis showed that IGF2BP3 overexpression reduced the apoptosis rate of AML cells, including early and late apoptosis. Importantly, IGF2BP3 overexpression successfully counteracted the apoptosis of AML cells induced by Jab1 deficiency (Supplemental Figure 14, C and F). These experimental findings collectively elucidate the role of the Jab1/IGF2BP3/LILRB4 axis in AML progression and immune evasion.

### Jab1 inhibitor CSN5i-3 impedes AML progression and extends survival.

**Figure 6:**
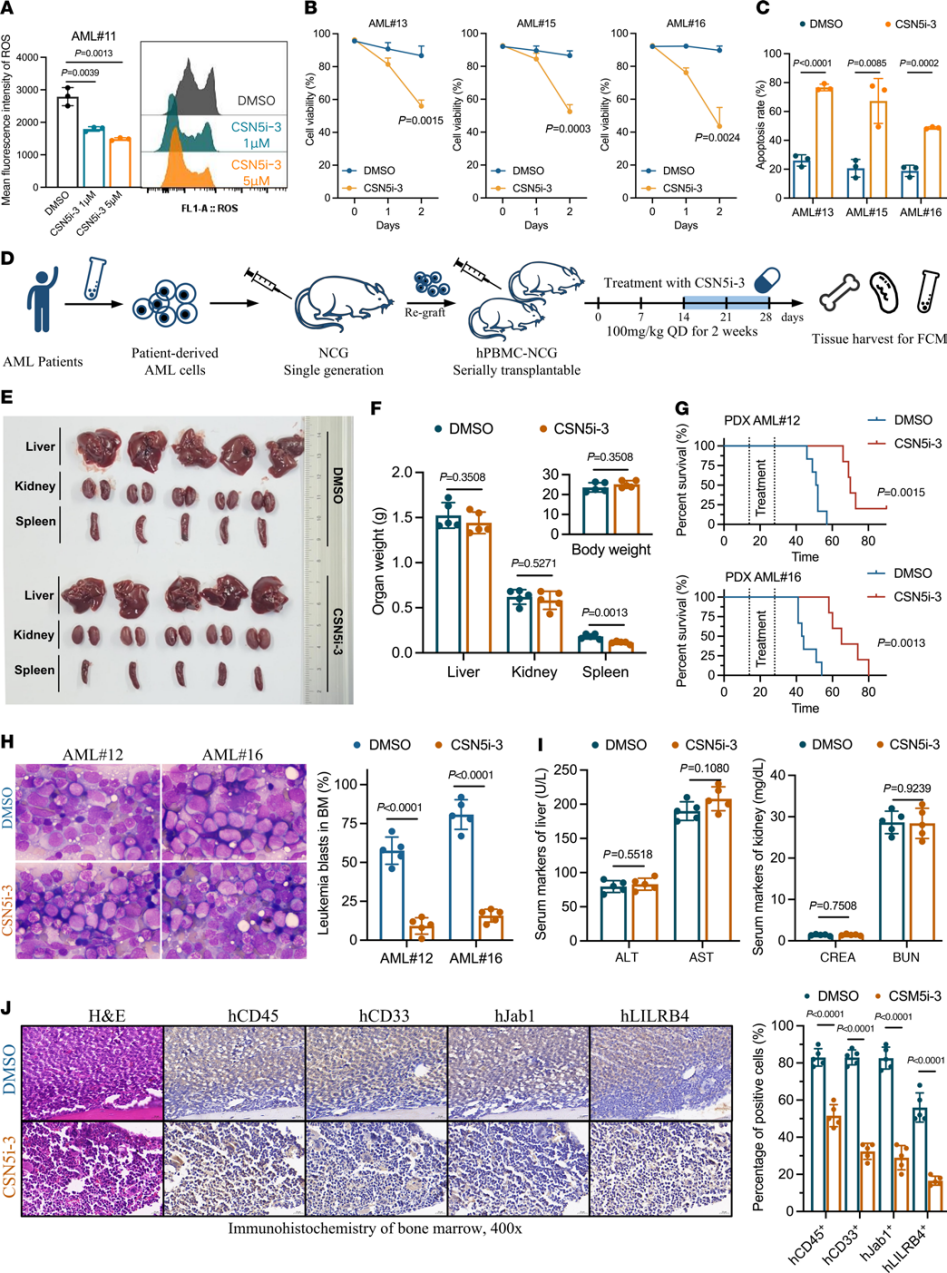
Jab1 inhibition by CSN5i-3 suppresses AML progression and prolongs survival in PDX models. (**A**) CSN5i-3 treatment reduces intracellular ROS levels in patient-derived AML cells (AML#11) in a dose-dependent manner, as assessed by flow cytometry. (**B** and **C**) CSN5i-3 significantly decreases cell viability and increases apoptosis in multiple primary AML samples (AML#13, #15, and #16) after 24–48 hours of treatment (*n* = 3 per group, by unpaired 2-tailed Student’s *t* test). (**D**) Schematic overview of the PDX model: patient-derived AML cells were transplanted into NCG mice and expanded for secondary xenografting, followed by CSN5i-3 treatment (100 mg/kg/d, 14 days). FCM, flow cytometry. (**E** and **F**) Gross organ examination and statistical analysis show reduced spleen size and weight in CSN5i-3–treated mice, with no significant changes in liver or kidney (*n* = 5 per group, by unpaired 2-tailed Student’s *t* test). (**G**) Kaplan-Meier survival curves indicate that CSN5i-3 treatment significantly prolongs survival in mice engrafted with AML#12 and AML#16 cells (Kaplan-Meier analysis with log-rank test). (**H**) Wright-Giemsa staining reveals reduced leukemic infiltration in bone marrow (BM) of CSN5i-3–treated mice (original magnification × 1,000; *n* = 5 per group, by unpaired 2-tailed Student’s *t* test). (**I**) Serum levels of liver (ALT and AST) and kidney (CREA and BUN) function markers remain unaffected by CSN5i-3 (*n* = 5 per group, by unpaired 2-tailed Student’s *t* test). ALT, alanine aminotransferase; AST, aspartate aminotransferase; CREA, creatinine; BUN, blood urea nitrogen. (**J**) Immunohistochemical staining of bone marrow further demonstrates reduced expression of human CD45, CD33, Jab1, and LILRB4 in CSN5i-3–treated mice (*n* = 5 per group, by unpaired 2-tailed Student’s *t* test).

Finally, we conducted functional analysis using patient-derived AML cells. Treatment with CSN5i-3 for 48 hours effectively reduced ROS levels in these cells, demonstrating its antioxidative properties (Figure 6A and Supplemental Figure 15, A and B). As anticipated, CSN5i-3 efficiently inhibited AML cell growth and promoted apoptosis (Figure 6, B and C). These findings strongly support the therapeutic potential of CSN5i-3 and emphasize the ability of Jab1 inhibitors to combat AML. Subsequently, we established a patient-derived xenograft (PDX) mouse model of AML to evaluate the antileukemic efficacy of CSN5i-3 in vivo. We intravenously transplanted human PBMCs (hPBMCs) into NCG mice, establishing a humanized mouse model with reconstituted human immune systems (hPBMC-NCG) (33, 34). Following hPBMC reconstitution, we observed a gradual increase in the proportion of human leukocytes in the peripheral blood of mice (Supplemental Figure 15, C–F). Secondarily expanded AML cells from patients were then reimplanted into hPBMC-NCG mice to successfully establish the PDX model. Oral gavage administration of CSN5i-3 at a dose of 100 mg/kg was initiated from days 14 to 28 after transplantation (Figure 6D). Initially, we assessed the potential in vivo toxicity of CSN5i-3. We observed no significant changes in mouse body weight or the weights of the liver, kidneys, or spleen (Figure 6, E and F). Measurements of alanine aminotransferase (ALT), aspartate aminotransferase (AST), creatinine (CREA), and blood urea nitrogen (BUN) levels also excluded the potential effects of CSN5i-3 on liver and kidney functions within this dose range (Figure 6I). Remarkably, mice treated with CSN5i-3 exhibited markedly prolonged survival, accompanied by a notable decrease in the proportion of leukemia blasts in the bone marrow and peripheral blood, as observed with Wright-Giemsa staining (Figure 6, G and H). Additionally, the population of hCD45^+^hCD33^+^ and hCD45^+^hLILRB4^+^ leukemia cells was markedly reduced in the CSN5i-3 treatment group (Supplemental Figure 15, G and H). Finally, consecutive sections of femoral tissue were immunohistochemically stained to observe the overlap between the positive areas of Jab1 and LILRB4 and those of hCD45^+^hCD33^+^ leukemia cells (Figure 6J). The severity of AML notably improved after CSN5i-3 treatment, indicating the anti-AML effect of the Jab1 inhibitor CSN5i-3 in vivo.

## Discussion

Immunotherapy is a promising research field in the current landscape of cancer treatment. The mechanisms underlying immunotherapy, particularly those involving T cell exhaustion and their reversal strategies, are pivotal for translating basic research into clinical applications (35). While advancements in novel therapies have improved the survival rates of patients with AML, the challenge of relapse persists, necessitating the exploration of new treatment strategies (3, 36). In this context, we pursued previous research to delve deeper into the association between oxidative stress and immune evasion. In this study, we identified small-molecule inhibitors capable of effectively disrupting the AML signaling network, thereby restoring cellular immune surveillance and impeding AML progression.

Oxidative stress induces DNA mutations within tumor cells and mediates the activation of proto-oncogenes or inactivation of tumor suppressor genes, leading to abnormal cell proliferation and tumor formation (37, 38). ROS also plays a crucial role in tumor development, particularly in immune regulation (38, 39). Elevated ROS levels are considered 1 of the primary factors contributing to immune suppression within the tumor microenvironment and inhibition of T cell activation (40). In the hematopoietic system, ROS play a vital role in balancing self-renewal and myeloid differentiation of HSCs. Disruption of this balance can result in leukemia (2, 40). In this study, we confirmed the significant role of Jab1 under oxidative stress conditions in immune evasion and malignant progression of AML. First, we observed that ROS levels in LSCs were significantly higher than those in normal hematopoietic stem progenitor cells. Based on this, we validated the increased expression of Jab1 under ROS induction and discovered that Jab1 depletion could inhibit the proliferation of primary human and murine AML cells in vivo and in vitro. Encouragingly, Jab1 depletion also demonstrated the ability to overcome AML immune evasion, with the deletion of Jab1 having no impact on normal hematopoietic function. These findings enhance our understanding of Jab1’s role in AML and may inform future development of therapeutic strategies targeting AML.

Reducing the expression of Jab1 in AML cells can alleviate the immunosuppressive microenvironment, whereas deleting Jab1 in hematopoietic cells does not affect the maturation and development of immune cells. We speculate that Jab1 does not directly affect the function of immune cells but rather regulates the expression of specific immune inhibitory targets in AML cells. Jab1, a transcriptional coactivator of AP-1/c-JUN, primarily exerts its oncogenic function by transcriptionally regulating downstream signaling networks (8, 41). Recent studies have identified LILRB4 as a novel immune inhibitory protein in leukemia, and our findings demonstrate that LILRB4 is regulated by Jab1. Although immune checkpoint inhibitors have shown promising results in various solid tumor treatments, their efficacy in leukemia is limited, suggesting unique mechanisms of leukemia evasion from treatment (42). Compared with normal hematopoietic cells, LILRB4 expression was significantly increased in leukemia cells, while the expression of PD-L1/PD-L2 remained relatively unchanged. These data partly explain why previous immunotherapy strategies targeting PD-1/PD-L1/PD-L2 have not shown significant efficacy in AML treatment (42). Chang et al. showed that anti-LILRB4 CAR T cells could selectively target monocyte-like AML cells without toxicity to normal hematopoietic progenitor cells (7). Additionally, the monoclonal antibody targeting LILRB4 (IO-202) from Immune-Onc Therapeutics has currently completed a phase I clinical trial (ClinicalTrials.gov, identifier: NCT04372433) to evaluate its safety and tolerability in AML with monocytic differentiation and chronic myelomonocytic leukemia (CMML), futher highlighting the importance of Jab1-mediated LILRB4 in T cell exhaustion and suggesting its potential as a therapeutic target.

However, our data indicated that Jab1 does not directly regulate LILRB4 transcription. Through a series of screenings, we identified IGF2BP3 as an RBP that stabilizes LILRB4 by mediating the m^6^A modification of LILRB4 mRNA. We found that Jab1 directly activated the transcription of IGF2BP3 by recruiting AP-1/c-JUN, thereby participating in the regulation of the AML signaling network. Recent evidence suggests that dysregulated m^6^A-related proteins and m^6^A modifications play crucial roles in the occurrence and progression of diseases such as cancer (31, 43–45). For example, the m^6^A methyltransferase METTL3 is highly expressed in AML, where it not only activates the oncogene c-MYC by enhancing SP1 m^6^A modification, but also promotes AML occurrence by regulating the translation of BCL2 and PTEN mRNA (42, 43). The m^6^A demethylases FTO and ALKBH5 are aberrantly expressed in AML and promote tumor occurrence and LSC self-renewal by m^6^A-dependent regulation of their target mRNAs (31, 42, 46–48). In our study, we found that IGF2BP3 participates in AML immune evasion by stabilizing the m^6^A-modified LILRB4 mRNA. However, the development of IGF2BP3 as a drug target faces challenges, including the absence of binding sites and limited functional detection methods. Fortunately, we discovered a small-molecule inhibitor, CSN5i-3, that regulates IGF2BP3 transcription and induces its overexpression by the upstream molecule Jab1. Using a PDX model derived from AML patients, this drug molecule effectively halted AML progression and prolonged patient survival, providing crucial insights for the development of novel treatment strategies against AML, particularly targeting the regulatory mechanisms of m^6^A modification and related proteins. Although oxidative stress is associated with elevated Jab1 expression and immune suppression in AML, our data suggest that the Jab1/IGF2BP3/LILRB4 axis functions downstream of ROS. The d-galactose model thus serves as a contextual reference rather than a mechanistic component. Further studies are needed to define the precise impact of ROS on this pathway.

Jab1 plays a multifaceted role in the regulation of transcription and translation (41). Zhu et al. emphasized the importance of PD-L1–mediated immune evasion in lung adenocarcinoma (49). However, our findings suggest that Jab1’s influence on PD-L1 expression in AML cells is relatively limited, indicating that Jab1 may exert distinct regulatory mechanisms in different malignancies. Notably, given the enrichment of LILRB4 expression in AML cases harboring specific molecular abnormalities, such as MLL rearrangements, the Jab1/IGF2BP3/LILRB4 axis may play a more prominent role in genetically defined AML subsets, although the subtype-specific functional significance of this pathway requires further investigation (50). Additionally, the protein structure of Jab1 contains a JAMM domain that is essential for ubiquitin-mediated protein degradation (51, 52). Increased levels of Jab1 can promote ubiquitination of NCoR, resulting in its degradation through the proteasome via its isopeptidase activity (53). NCoR plays a crucial role as a coactivator in the suppression of estrogen receptor α (ERα) target genes, particularly in the context of ERα- and tamoxifen-induced pathways in breast cancer models, which is essential for the development of tamoxifen resistance (53). Therefore, a thorough investigation of Jab1 mechanisms in various settings is necessary to comprehend its complex involvement in cellular physiology and disease progression.

In conclusion, our study presents a detailed investigation of Jab1’s role in AML progression and its ability to evade immune responses through the activation of downstream signaling pathways, supported by evidence from both in vitro and in vivo experiments. Furthermore, our analysis of the interplay among oxidative stress, RNA epigenetic modifications, and immune regulation sheds light on their mechanisms in AML. These findings support the development of potential therapeutic strategies and have significant clinical implications in the treatment of leukemia.

## Methods

### Sex as a biological variable.

Bone marrow aspirates were collected from newly diagnosed AML patients at the Department of Hematology, Zhongnan Hospital of Wuhan University, with written informed consent obtained from all participants in accordance with institutional ethical guidelines. The study cohort included both male and female patients; detailed clinical characteristics including age and sex distribution are provided in Supplemental Table 1.

The C57BL/6-Jab1-flox (strain T005082), B6-Rag1-KO (strain T004753), NCG (strain T001475), and WT C57BL/6 (strain N000013) mice used in this study were purchased from GemPharmatech Co. Ltd. B6.Cg-Tg (Mx1-Cre)1Cgn/J and C57BL/6-CD45.1-Ptprc mice were provided by Haojian Zhang’s research team at the School of Medicine, Wuhan University (17, 46). To generate conditional knockout mice, Jab1^fl/null^ heterozygous male and female mice were crossed to obtain Jab1^fl/fl^ homozygous mice. These Jab1^fl/fl^ mice were crossed with adult Mx1-Cre mice to produce Jab1^fl/null^ Mx1-Cre genotype mice. Subsequently, adult Jab1^fl/null^ Mx1-Cre mice were bred with the Jab1^fl/fl^ mice. Genotyping of the offspring was performed using PCR amplification of the excision site. This breeding strategy resulted in the generation of Jab1^fl/null^ Mx1-Cre (Jab1-HET) and Jab1^fl/fl^ Mx1-Cre (Jab1-KO) mice. Upon reaching 4 weeks of age, mice were intraperitoneally injected with poly(I:C) (catalog TLRL-PIC-5; InvivoGen) at a dose of 5 mg/kg for a total of 3 injections, administered every other day, to induce deletion of the Jab1 gene. The mice were maintained under a 12-hour light/12-hour dark cycle with unrestricted access to food and water. Random allocation of mice was performed across the experimental groups. All sexes of mice were included in the study.

### Cell culture.

MV411 (ATCC, CRL-9591; RRID: CVCL_0064), HL60 (ATCC, CCL-240; RRID: CVCL_A794), and K562 (ATCC, CCL-243; RRID: CVCL_0004) cells were cultured in IMDM with 10% FBS and 1% penicillin-streptomycin. MOLM13 (DSMZ, ACC-554; RRID: CVCL_2119) and Kasumi-1 (ATCC, CRL-2724; RRID: CVCL_0589) cells were maintained in RPMI-1640 with 10% FBS and 1% penicillin-streptomycin. THP1 (ATCC, TIB-202; RRID: CVCL_0006) cells were cultured in RPMI-1640 with 10% FBS, 0.05 mM β-mercaptoethanol, and 1% penicillin-streptomycin. C1498 (ATCC, TIB-49; RRID: CVCL_3494) cells were grown in high-glucose DMEM with 10% FBS and 1% penicillin-streptomycin. Primary patient cells were cultured in IMDM with 20% FBS and 10 ng/mL SCF, TPO, IL-3, and IL-6 (PeproTech). All cells were maintained at 37°C in a 5% CO_2_ incubator. Cells were passaged at 0.5× 10^6^ to 1 × 10^6^ cells/mL, collected, centrifuged at 200*g* for 5 minutes, resuspended in fresh medium, and transferred to new flasks.

### RT-qPCR.

Total RNA was extracted from cells using TRIzol reagent (catalog 9109; Takara) following the manufacturer’s protocol. Subsequently, cDNA was synthesized using a PrimeScript RT Master Mix Kit (catalog RR037A; Takara). RT-qPCR analysis was conducted using a QuantiTect SYBR Green PCR kit (catalog CW0957; CWBIO). Expression levels were normalized to the housekeeping controls GAPDH or Actin. The primers used for amplification are listed in Supplemental Table 3.

### Immunoblot assay.

The gel was prepared using a PAGE Gel Rapid Preparation Kit (Enzyme Organism), and proteins of different molecular weights were separated by electrophoresis. The proteins were transferred from the gel to a PVDF membrane using a wet transfer method. PVDF membranes were blocked with 5% skim milk and incubated at 37°C for 1 hour. Subsequently, the membranes were incubated with primary antibodies specific to the target proteins, followed by HRP-conjugated secondary antibodies. Protein bands were visualized using freshly prepared ECL solution in a chemiluminescence imaging system. Details of all antibodies used, including clone ID, source, and catalog numbers, are provided in Supplemental Tables 4 and 5.

### Immunohistochemistry.

Mouse bone marrow was harvested for immunohistochemistry to assess the AML burden. Briefly, the stripped tibia or femur was fixed in 4% paraformaldehyde and decalcified in EDTA reagent for 2 weeks before being embedded in paraffin. Longitudinal sections (4 μm) of the tibia or femur were prepared and subjected to antigen retrieval. Nonspecific staining was blocked with BSA at room temperature for 30 minutes. Subsequently, the sections were incubated with anti-hCD45/hCD33/hJab1/hLILRB4 antibodies overnight at 4°C. The corresponding secondary antibody was applied to the tissues for 50 minutes at room temperature. The peroxidase reaction was visualized using a DAB peroxidase substrate. After counterstaining with hematoxylin, the slides were dehydrated, mounted, and observed under an optical microscope.

### Cell proliferation, apoptosis, and cell cycle assays.

Cell proliferation assays were conducted using the Cell Counting Kit-8 (CCK-8; catalog CA1210; Solarbio). Proliferation rates were assessed at 0, 24, 48, 72, and 96 hours after infection, with absorbance readings taken at 450 nm, according to the manufacturer’s instructions. Apoptosis was determined by flow cytometry using an annexin V-FITC/PI apoptosis detection kit (catalog AP101; MultiSciences). Cell cycle analysis was performed using flow cytometry staining with Ki-67 and Hoechst 33342 (catalog B2261; Sigma-Aldrich) to distinguish the G0, G1, S, and G2-M phases. All flow cytometry data were compensated using single-stained controls. For each sample, at least 10,000 singlet events were collected. A consistent gating strategy was applied across all experimental groups. Data acquisition was performed on a FACSCanto II flow cytometer (BD Biosciences), and analyses were conducted using FlowJo software.

### RNA stability assay.

The method for the RNA stability assay has been described previously (5, 46). Cells were seeded in 6-well plates and treated with 5 μg/mL actinomycin D (catalog A9415; Sigma-Aldrich) for 0, 2, 4, and 6 hours. After treatment at different time points, total RNA was collected from the cells and subjected to RT-qPCR to quantify the relative expression of LILRB4 mRNA (relative to 0 hour). The degradation curves were fitted to calculate the mRNA half-life.

### High-throughput sequencing and data analysis.

RNA-Seq was conducted using total RNA extracted from cells in the logarithmic growth phase, which was used to construct RNA-Seq libraries by LC Biotech Inc. The process included mRNA isolation, fragmentation, double-stranded cDNA synthesis, chemical modification, magnetic bead purification, fragment selection, and library amplification for sequencing on the Illumina platform.

MeRIP-Seq was conducted as described, with assistance from the School of Chemistry, Wuhan University (54). Briefly, total RNA was fragmented into approximately 100 nt fragments with zinc acetate. Then, fragmented RNA was immunoprecipitated with m^6^A antibodies. Nonspecific binding was removed through a stringent washing process. Bound m^6^A RNA was eluted by competition. For input RNA, ribosomal RNA was removed. Libraries for both immunoprecipitated and input RNAs were prepared accordingly.

### RIP assay.

The RIP assay was conducted following the protocol provided with the RIP Kit (catalog P0102; Geneseed). In summary, magnetic beads were first combined with anti-m^6^A/IGF2BP3/IgG antibodies, followed by addition of cell lysates. Subsequently, the bound complexes were thoroughly washed, eluted, purified, and analyzed using RT-qPCR. The enrichment of the precipitated RNAs was normalized to the input controls.

### ChIP assay.

ChIP assays were conducted using a ChIP assay kit (catalog 56383S; Cell Signaling Technology) following the manufacturer’s protocol. In brief, cells were collected and fixed for 10 minutes at 37°C with 1% formaldehyde, followed by sequential steps, including SDS lysis and DNA shearing, immunoprecipitation of protein-DNA complexes, reversal of cross-linked DNA, and DNA purification. Subsequently, the immunoprecipitated DNA fragments were subjected to PCR for detection. Normal rabbit IgG was used as the negative control.

### Generation of leukemia mouse models.

The MLL-AF9–induced AML mouse model has been previously described (46, 55). Briefly, lineage-negative (Lin^−^) bone marrow cells from C57BL/6J mice were enriched and transduced in vitro with MSCV-MLL-AF9-GFP retrovirus. For Jab1-knockdown experiments, MLL-AF9–transduced cells were further infected with a GFP-labeled lentiviral vector encoding either shJab1 or control shRNA. A total of 5 × 10^4^ GFP^+^ cells were transplanted via tail vein into lethally irradiated WT C57BL/6 recipient mice (900 cGy, split dose). Primary recipient mice were monitored for disease progression over a 28-day period or until moribund. For secondary transplantation, 1 × 10^6^ GFP^+^ leukemia cells harvested from primary bone marrow were injected into sublethally irradiated (450 cGy) secondary recipients. Survival and leukemia burden were assessed at humane endpoints.

In parallel, to generate genetically engineered Jab1-deficient AML models, Lin^−^ bone marrow cells from Jab1^fl/fl^ Mx1-Cre mice were transduced with MSCV-MLL-AF9-GFP and transplanted into lethally irradiated recipients. Poly(I:C) was administered every other day (3 doses total) beginning on day 7 after transplant to induce Cre recombinase and delete Jab1 in hematopoietic cells.

### Competitive reconstruction assay.

From donor mice aged between 6 and 8 weeks, 5 × 10^6^ bone marrow cells were isolated from WT, Jab1-HET, and Jab1-KO mice and then mixed with 5 × 10^6^ competitive cells from CD45.1^+^ mice at a 1:1 ratio. The mixed cells were injected via the tail vein into lethally irradiated CD45.1^+^ recipient mice. The cell engraftment capacity and the status of multiple lineage cells from donor mice were analyzed at 4, 8, 12, and 16 weeks after transplantation. After 16 weeks, the proportions of donor bone marrow stem cells and progenitor cells were analyzed.

### Mouse in vivo imaging.

After establishing a mouse AML model using leukemia cells transfected with the luciferase reporter gene, imaging sessions were conducted weekly. The procedure involved booting and initializing the computer according to the instructions provided by the imaging system. Isoflurane was loaded into the anesthesia pump using a syringe, and IVIS oxygen was connected. Each mouse received an intraperitoneal injection of 150 mg/kg d-luciferin potassium (catalog 115144-35-9; MeilunBio). After approximately 5 minutes of anesthesia, the mouse was carefully transferred to a dark box, positioned with its back facing upward, head toward the air vent, and limbs and tail properly arranged. Imaging settings were adjusted as required, and luminescence and exposure times were selected for imaging.

### Bone marrow hematopoietic population analysis.

Multicolor analysis of murine HSPCs was performed according to the OMIP-059 protocol (56). Using a full-spectrum flow cytometer, we analyzed the specific expression patterns of the cell surface proteins CD16/32, Sca-1, c-Kit, CD150, CD48, CD127, CD34, and CD135 to identify murine bone marrow HSCs and 4 types of multipotent progenitor (MPP): common myeloid progenitor (CMP), granulocyte-macrophage progenitor (GMP), megakaryocyte-erythroid progenitor (MEP), and common lymphoid progenitor (CLP). The details are as follows: HSCs, Lin^–^Sca-1^+^c-Kit^+^CD150^+^CD48^–^CD34^–^CD135^–^; MPP1, Lin^–^Sca-1^+^c-Kit^+^CD150^+^CD48^–^CD34^+^CD135^–^; MPP2, Lin^–^Sca-1^+^c-Kit^+^CD150^+^CD48^+^; MPP3, Lin^–^Sca-1^+^c-Kit^+^CD150^–^CD48^+^CD135^–^; MPP4, Lin^–^Sca-1^+^c-Kit^+^CD150^–^CD48^+^CD135^+^; MPP5, Lin^–^Sca-1^+^c-Kit^+^CD150^–^CD48^–^; CMP, Lin^–^Sca-1^–^c-Kit^+^CD34^+^CD16/32^–^; GMP, Lin^–^Sca-1^–^c-Kit^+^CD34^+^CD16/32^+^; MEP, Lin^–^Sca-1^–^c-Kit^+^CD34^–^CD16/32^–^; and CLP, Lin^–^Sca-1^lo^c-Kit^lo^CD135^+^CD127^+^. To distinguish between CD45.1/CD45.2 donor/recipient-derived peripheral blood cells and bone marrow cells, anti-CD45.1 and anti-CD45.2 staining was required. Detailed information regarding the antibodies used is provided in Supplemental Table 4. Flow cytometry was performed on a Cytek Aurora/NL full-spectrum flow cytometer. At least 4 million total bone marrow events were acquired per sample to ensure accurate resolution of rare stem and progenitor populations.

### Immune cell population analysis.

Subpopulation analysis of mouse immune cells was performed following the OMIP-079 protocol (20). Using a full-spectrum flow cytometer, we analyzed the specific expression patterns of cell surface proteins CD3, CD4, CD8, CD44, and CD62L to identify distinct subpopulations of mouse immune cells, including T_N_ cells, central memory T (T_CM_) cells, T_EM_ cells, and T_EMRA_ cells: CD4^+^ T_N_, CD3^+^CD4^+^CD8^–^CD44^int/lo^CD62L^+^; CD4^+^ T_CM_, CD3^+^CD4^+^CD8^–^CD44^hi^CD62L^+^; CD4^+^ T_EM_, CD3^+^CD4^+^CD8^–^CD44^hi^CD62L^–^; CD4^+^ T_EMRA_, CD3^+^CD4^+^CD8^–^CD44^int/lo^CD62L^–^; CD8^+^ T_N_, CD3^+^CD8^+^CD4^–^CD44^int/lo^CD62L^+^; CD8^+^ T_CM_, CD3^+^CD8^+^CD4^–^CD44^hi^CD62L^+^; CD8^+^ T_EM_, CD3^+^CD8^+^CD4^–^CD44^hi^CD62L^–^; and CD8^+^ T_EMRA_, CD3^+^CD8^+^CD4^–^CD44^int/lo^CD62L^–^. To analyze the proliferation and cycling of immune cell subsets, Hoechst 33342 and APC-Ki67 staining was also required. Flow cytometry was conducted on a Cytek Aurora/NL full-spectrum cytometer. For each sample, a minimum of 500,000 total events were acquired to ensure robust resolution of low-frequency subsets. Detailed information regarding the antibodies used is provided in Supplemental Table 3.

### PDX model establishment.

hPBMCs were intravenously injected into NCG-Prkdc^KO/KO^~Il2rg^KO/KO^ (NCG) mice to reconstitute the human immune system. Flow cytometry was used to assess the levels and functionality of human immune cells in mice, confirming the establishment of a humanized immune system denoted as hPBMC-NCG. Leukemic cells derived from patients with AML were cultured, and logarithmically growing cells were harvested and centrifuged at 500*g* for 5 minutes. A suspension containing 1 × 10^7^ cells in 200 μL PBS was intravenously administered to recipient mice irradiated with sublethal doses, thus establishing a PDX mouse model. Following its successful establishment, the antileukemia effects and safety profile of the Jab1 inhibitor CSN5i-3 (catalog 2375740-98-8; MedChemExpress) were evaluated in vivo, with the survival of all mice as the primary endpoint, while monitoring leukemia progression.

### Statistics.

Statistical analyses were performed using R 4.2.2 and GraphPad Prism (version 8.3.0). Two-tailed Student’s *t* test and 1-way ANOVA were used to compare 2 and multiple means, respectively. A χ^2^ test was used for qualitative analysis. Survival curves were plotted using the Kaplan-Meier method, and survival data were assessed using the log-rank test. Both univariate and multivariate Cox regression analyses were conducted, with significance set at *P* < 0.05. FlowJo (version 10.8.1) was used for the analysis and visualization of flow cytometry–related data.

### Study approval.

The human studies received approval from the Ethics in Research Committee of Zhongnan Hospital of Wuhan University (protocol number 2022118), and written informed consent was obtained from participants. All animal experiments were conducted in compliance with institutional guidelines and were approved by the Wuhan University IACUC (protocol numbers BZ20220015 and WP20230121).

### Data availability.

The bioinformatics analysis in this study utilized high-throughput sequencing data from public databases including The Cancer Genome Atlas (TCGA), Therapeutically Applicable Research to Generate Effective Treatment (TARGET), Gene Expression Omnibus (GEO), and the Oregon Health and Science University (OHSU) AML cohort. Specifically, GEO datasets GSE37642, GSE13159, GSE30285, GSM935411, GSE37642, GSE71014, GSE30029, GSE83533, and GSE6891 were included. The Cancer Cell Line Encyclopedia database was used for gene expression analysis of various AML cell lines. Additionally, the sequencing data generated directly from this study were submitted to the GEO repository under the accession number GSE254292. The details of the detection reagents are provided in Supplemental Table 5. The gene set linked to oxidative stress originated from the Gene Ontology Biological Process “response to oxidative stress” gene set in the MSigDB database (https://www.gsea-msigdb.org/gsea/msigdb). All other data that support the findings of this study are presented in the main paper and in the supplemental tables and figures. Values for all data points in graphs are reported in the Supporting Data Values file. Raw immunoblot data are reported in the full unedited blot and gel images file.

## Author contributions

NZ, QW, and FZ conceived and supervised the study. GC, LL and ZW designed the methodology. GC and ZW performed the formal analysis. LM, YL, and JW curated the data. NZ QW, GC, and FZ drafted the manuscript. NZ, QW, FZ, and X. Liu reviewed and edited the manuscript. X. Li and X. Liu contributed to data visualization. NZ and FZ provided resources and acquired funding. FZ was responsible for project administration. All authors participated in the investigation and approved the final version of the manuscript.

## Supplementary Material

Supplemental data

Unedited blot and gel images

Supporting data values

## Version 1 08/05/2025

In-Press Preview

## Version 2 10/15/2025

Electronic publication
