# Supplementary material for: Jab1 promotes immune evasion and progression in acute myeloid leukemia models under oxidative stress
Source: J Clin Invest. 2025 Aug 5;135(20):e183761. doi: 10.1172/JCI183761 (PMC12520683; doi:10.1172/JCI183761)
Supplement: Supplemental data [file jci-135-183761-s019.pdf]

Supplemental Data

Jab1 promotes immune evasion and progression in acute myeloid leukemia models under oxidative stress

Nan Zhang[1,2,#], Qian Wang[1,#], Guopeng Chen[1], Li Liu[1], Zhiying Wang[4], Linlu Ma[1], Yuxing Liang[1], Jinxian Wu[1], Xinqi Li[1], Xiaoyan Liu[1], Fuling Zhou[1,3,*]

[1]Department of Hematology, Zhongnan Hospital of Wuhan University, Wuhan, Hubei, China.

[2]Department of Hematology, The Second Affiliated Hospital of Chongqing Medical University, 76 Linjiang Road, Chongqing, China.

[3]Research Center for Lifespan Health, Wuhan University, Wuhan, Hubei, China.

[4]College of Chemistry and Molecular Sciences, Wuhan University, Wuhan 430072, China.

[#]These authors have contributed equally to this work.

[*]Correspondence: Fuling Zhou, Department of Hematology, Zhongnan Hospital of Wuhan University, No.169 Donghu Road, Wuhan, 430072 China. Email: zhoufuling@whu.edu.cn.

Running Title: Jab1 Regulates Immune Evasion in AML

Catalogue:

Supplementary Table S1 1

Supplementary Table S2 2

Supplementary Table S3 3

Supplementary Table S4 5

Supplementary Table S5 8

Supplementary Figure S1 11

Supplementary Figure S2 13

Supplementary Figure S3 15

Supplementary Figure S4 17

Supplementary Figure S5 19

Supplementary Figure S6 20

Supplementary Figure S7 22

Supplementary Figure S8 24

Supplementary Figure S9 25

Supplementary Figure S10 27

Supplementary Figure S11 29

Supplementary Figure S12 31

Supplementary Figure S13 33

Supplementary Figure S14 35

Supplementary Figure S15 37

Supplementary Table S1

Genetic background of patients with acute myeloid leukemia.

Sample_ID	Patient_ID	Age	Sex	Category	Karyotype	Genetic_alterations	Disease_status
AML#1	DDZ	70	Female	AML-M2	Normal	NPM1(+), TET2(+)	Newly diagnosed
AML#2	LQP	55	Male	AML-M5	Normal	FLT3-ITD(+),NPM1(+), DNMT3A(+)	Relapsed/ Refractory
AML#3	MJL	61	Male	AML-M5	Normal	N/A	N/A
AML#4	HT	22	Male	AML-M4	inv(16)/t(16;16)	N/A	Newly diagnosed
AML#5	ZCS	52	Male	AML-M2	Normal	IDH2(+), DNMT3A(+), SRSF2(+)	Relapsed/ Refractory
AML#6	YAH	52	Female	AML-M2	Normal	CEBPA(+)	Newly diagnosed
AML#7	FH	23	Female	AML-M2	Trisomy 8	N/A	Newly diagnosed
AML#8	GGY	79	Female	AML-M2	N/A	FLT3-ITD(+),NRAS(+), U2AF1(+)	N/A
AML#9	WWZ	52	Female	AML-M1	Normal	FLT3-ITD(+),CEBPA(+)	N/A
AML#10	YS	49	Female	AML-M4	Complex karyotype	ZRSR2(+)	Relapsed/ Refractory
AML#11	DCJ	82	Male	AML-M2	Complex karyotype	RUNX1(+), TP53(+), IDH2(+), SRSF2(+)	N/A
AML#12	LJ	46	Male	AML-M5	N/A	FLT3-ITD, TP53(+), IDH2(+)	Relapsed/ Refractory
AML#13	ZZJ	67	Female	AML-M5	Normal	IDH1(+)	N/A
AML#14	DFL	44	Female	AML-M4	Normal	FLT3-TKD(+)	N/A
AML#15	ZSJ	66	Female	AML-M1	Complex karyotype	FLT3-ITD(+), NPM1(+), TET2(+), ASXL1(+), U2AF1(+)	N/A
AML#16	LJA	52	Male	AML-M2	Normal	FLT3-ITD(+)	Newly diagnosed

Supplementary Table S2

Univariate and multivariate analysis of Jab1 expression for overall survival in patients with AML from TARGET cohort.

	Univariate analysis			Multivariate analysis		
Variable	HR	95%CI	P-value	HR	95%CI	P-value
**Gender** (male vs.female)	1.05	0.71–1.56	0.9123			
**WBC** (>50*10^9/L vs.<=50*10^9/L)	1.39	0.88–2.21	0.0877	1.03	0.64-1.87	0.1156
**BM_blast** (>50% vs.<=50%)	1.12	0.78–1.8	0.1197			
**FLT3-ITD** (positive vs.negative)	1.61	1.15–2.32	**0.0454**	1.39	0.84-2.31	0.0931
**RUNX1-RUNX1T1** (positive vs.negative)	0.57	0.24–1.14	0.122			
**WT1** (mutated vs. wild type)	1.73	0.73–3.09	0.722			
**Risk_Cyto** (poor vs. intermediate/good)	2.93	1.48-5.83	**0.0007**	2.66	1.28-4.6	**0.0073**
**Complex_Cyto** (yes vs. no)	1.89	1.09-3.17	**0.0151**	1.61	0.8-3.11	0.0662
**Jab1_expression** (high vs. low)	1.93	1.38-2.94	**0.0013**	1.59	1.14-2.46	**0.0388**

Abbreviations: WBC, white blood cell; BM, bone marrow; PB, peripheral blood; HR, hazard ratio; CI, confidence interval;

Variables with P<0.1 in the univariate analysis were included in the multivariate analysis.

Supplementary Table S3
List of oligonucleotides.

Name	Sequence	Note
Human-ACTIN-F	CGTGGACATCCGCAAAG	qPCR primers
Human-ACTIN-R	AAGGTGGACAGCGAGGC	
Human-GAPDH-F	CAGGAGGCATTGCTGATGAT	
Human-GAPDH-R	GAAGGCTGGGGCTCATTT	
Human-Jab1-F	CGGTATGGCCCAGAAAACCT	
Human-Jab1-R	CTTCCAAGTTGCCTCCCGAT	
Human-IGF2BP3-F	GGGAGGTGCTGGATAGTTTAC	
Human-IGF2BP3-R	CTAGCTTGGTCCTTACTGGAATAG	
Human-PDL1-F	AATTGGTCATCCCAGAACTACCTCT	
Human-PDL1-R	TCATTCTCCCTTTTCTTAAACGGA	
Human-PDL2-F	CATTGACCTTCAAAGTCAGATGGA	
Human-PDL2-R	TTAGGGCTATCACTGTGGCTATGA	
Human-LILRB1-F	CTGTTACTATGGTAGCGACACTG	
Human-LILRB1-R	CACACTGGAGGATTACATTCCC	
Human-LILRB2-F	GCATCTTGGATTACACGGATACG	
Human-LILRB2-R	CTGACAGCCATATCGCCCTG	
Human-LILRB3-F	GAACACCAGCTCCCCCGGAC	
Human-LILRB3-R	GGACAGGGCCCTGCAGGG	
Human-LILRB4-F	CATCCATGACAGAGGACTATGC	
Human-LILRB4-R	GGGCTGAAAGGGTGGGTTTA	
Human-LILRB5-F	CAGCCTCTGTGATAGCTCGG	
Human-LILRB5-R	ACCGTGGATGGAATGTGGAAC	
Mouse-Jab1-F	TTGCATCTTGATTGTGGAGCGAC	
Mouse-Jab1-R	CAGTATTTAAAGTAGTGGTGATCC	
Mouse-GAPDH-F	CATCACTGCCACCCAGAAGACTG	
Mouse-GAPDH-R	ATGCCAGTGAGCTTCCCGTTCAG	
Human-LILRB4-Primer 1-F	AGTGACCCAAAAGGATACGGA	ChIP-qPCR primers
Human-LILRB4-Primer 1-R	GAAGGAGAGGCTTGCAGGAG	
Human-LILRB4-Primer 2-F	ACGTGCCCACTCAAATGGAT	
Human-LILRB4-Primer 2-R	ACAAGCAAGCAAAAAGGCCA	
Human-LILRB4-Primer 3-F	TTATGGGGTACAATGTGTTATAAATGT	
Human-LILRB4-Primer 3-R	CTGGGGTTACAGGCATGAGC	
Human-LILRB4-Primer 4-F	ACACAGGAGGCTAAAGCAGG	
Human-LILRB4-Primer 4-R	ATGCAGTCTCACTCTGTCGC	
Human-LILRB4-Primer 5-F	AGGATCTTGGGTTGGGGAGA	
Human-LILRB4-Primer 5-R	CAAAGCCACCAGTCCCGTTA	
Human-IGF2BP3-Primer 1-F	ATGTTGGATTCGCTTGGCAG	
Human-IGF2BP3-Primer 1-R	TGTAATTTTGTGTTAAGGAACCGGA	
Human-IGF2BP3-Primer 2-F	CTGACACCTTCCGGTTCCTT	
Human-IGF2BP3-Primer 2-R	TGAATGGTAGACTTAAACATGGGA	
Human-IGF2BP3-Primer 3-F	TTGACTTCTCTCCTCCAGCG	

Human-IGF2BP3-Primer 3-R	TTGCGCGATTCAGAAAAGGC	
Human-IGF2BP3-Primer 4-F	GCCTGTTAGTACCTCTGCCC	
Human-IGF2BP3-Primer 4-R	CTGACGGTCACAACTCGAGT	
Human-LILRB4-Primer 6-F	GGACAGACAGAACCCACTGG	RIP/m^6A-qPCR primers
Human-LILRB4-Primer 6-R	CTGTCATCACCAGCTCCAGG	
shJab1#1	CTACAAACCTCCTGATGAA	shRNA target oligo
shJab1#2	GGACTAAGGATCACCATTA	
shJab1#3	GTCTCAGGTTATTAAGGATAA	
shIGF2BP3#1	GCACATTTAATTCCTGGATTA	
shIGF2BP3#2	GCAGGAATTGACGCTGTAT	
Mouse-Jab1-flox5-F1	TAATCTCAGCAGAGGTGGGTGATC	Genotype identification of mice
Mouse-Jab1-flox5-R1	CTGCTGAGGTGAGGGAGTAATGAT	
Mouse-Jab1-flox3-F1	CTGCAAGTGTAGGTCATCTCAGG	
Mouse-Jab1-flox3-R1	ACAGGTACAGCTCAGGGGTAGAGT	
Mouse-Mx1-Cre-1	CGGTTATTCAACTTGCACCA	
Mouse-Mx1-Cre-2	GACAAAATGGTGAAGGTCGG	
Mouse-Mx1-Cre-3	CAAAGGCGGAGTTACCAGAG	
Mouse-Mx1-Cre-4	GTGAGTTTCGTTTCTGAGCTCC	

Supplementary Table S4
List of resources for antibodies.

Antibodies	Clone ID	Source	Identifier
Anti-GAPDH antibody	1E6D9	Proteintech	Cat# 60004-1-Ig; RRID: AB_2107436
Anti-Tubulin antibody	N/A	Proteintech	Cat# HRP-66031; RRID: AB_2687491
Anti-LMNB1 antibody	N/A	Proteintech	Cat# 12987-1-AP; RRID: AB_2136290
Anti-LILRB4 antibody	N/A	Proteintech	Cat# 28422-1-AP; RRID: AB_3086050
Anti-IGF2BP3 antibody	N/A	Proteintech	Cat# 14642-1-AP; RRID: AB_2122782
Anti-Jab1 antibody	B-7	Santa Cruz Biotechnology	Cat# sc-13157; RRID: AB_627835
Anti-c-JUN antibody	60A8	Cell Signaling Technology	Cat# 9165S; RRID: AB_2130165
HRP-conjugated Goat Anti-Mouse IgG(H+L)	N/A	Proteintech	Cat# SA00001-1; RRID: AB_2722565
HRP-conjugated Goat Anti-Rabbit IgG(H+L)	N/A	Proteintech	Cat# SA00001-2; RRID: AB_2722564
Anti-human CD279 (PD-1) APC	A17188A	BioLegend	Cat# 379207; RRID: AB_2922606
Anti-human CD366 (Tim-3) PE	A18087E	BioLegend	Cat# 364805; RRID: AB_2922577
Anti-human CD274 FITC	MIH2	BioLegend	Cat# 393605; RRID: AB_2734471
Anti-human CD273 PE	24F.10C12	BioLegend	Cat# 329605; RRID: AB_1089021
Anti-human CD4 FITC	M-T477	BD Biosciences	Cat# 556615; RRID: AB_396487
Anti-human CD8 PerCP/Cyanine5.5	SK1	BD Biosciences	Cat# 565310; RRID: AB_2687497
Anti-human CD85k (ILT3) APC	ZM4.1	BioLegend	Cat# 333015; RRID: AB_2565560
Anti-Human CD45 FITC	HI30	BioLegend	Cat# 304054; RRID: AB_2564154
Anti-human CD33 APC	WM53	BD Biosciences	Cat# 551378; RRID:AB_398502
Anti-human CD3 APC/Cyanine7	SK7	BD Biosciences	Cat# 557832; RRID:AB_396890
Anti-mouse CD3 APC/Cyanine7	17A2	BioLegend	Cat# 100222; RRID: AB_2242784

Anti-mouse CD4 Brilliant Violet 510™	RM4-5	BioLegend	Cat# 100559; RRID: AB_2562608
Anti-mouse CD8a PerCP/Cyanine5.5	53-6.7	BioLegend	Cat# 100734; RRID: AB_2075238
Anti-mouse/human CD44 Brilliant Violet 605™	IM7	BioLegend	Cat# 103047; RRID: AB_2562451
Anti-mouse CD62L PE/Cyanine7	MEL-14	BioLegend	Cat# 104418; RRID: AB_313103
Anti-mouse Ki-67 APC	16A8	BioLegend	Cat# 652406; RRID: AB_2561930
Anti-mouse CD223 (LAG-3) Brilliant Violet 421™	C9B7W	BioLegend	Cat# 125221; RRID: AB_2572080
Anti-mouse CD85k (gp49 Receptor) PE	H1.1	BioLegend	Cat# 144903; RRID: AB_2561653
Anti-mouse CD117 (c-kit) PE/Cyanine7	2B8	BioLegend	Cat# 105814; RRID: AB_313223
Anti-mouse Ly-6G/Ly-6C (Gr-1) Brilliant Violet 605™	RB6-8C5	BioLegend	Cat# 108439; RRID: AB_2562333
Anti-mouse/human CD11b FITC	M1/70	BioLegend	Cat# 101205; RRID: AB_312788
Anti-mouse/human CD11b APC	M1/70	BioLegend	Cat# 101212; RRID: AB_312795
Anti-mouse TER-119 PE/Cyanine7	TER-119	eBioscience	Cat# 25-5921-82; RRID: AB_469661
Anti-mouse/human CD45R(B220) APC	RA3-6B2	eBioscience	Cat# 17-0452-83; RRID: AB_469396
Anti-mouse CD135 PE	A2F10	BioLegend	Cat# 135305; RRID: AB_1877218
Anti-mouse CD16/32 PerCP/Cyanine5.5	93	BioLegend	Cat# 101323; RRID: AB_1877268
Streptavidin APC/Cyanine7	N/A	BD Biosciences	Cat# 554063; RRID:AB_10054651
Anti-mouse CD34 Brilliant Violet 421™	SA376A4	BioLegend	Cat# 152208; RRID: AB_2650766
Anti-mouse CD127 Brilliant Violet 510™	A7R34	BioLegend	Cat# 135033; RRID: AB_2564576
Anti-mouse Sca-1 Brilliant Violet 605™	D7	BioLegend	Cat# 108134; RRID: AB_2650926
Anti-mouse CD150 Brilliant Violet 785™	TC15-12F12.2	BioLegend	Cat# 115937; RRID: AB_2565962
Anti-mouse CD48 Pacific Blue	HM48-1	BioLegend	Cat# 103418; RRID: AB_756140

Anti-mouse CD45.1 PE-CF594	N/A	BD Biosciences	Cat# 562452; RRID: AB_11152958
Anti-mouse CD45.2 Alexa Fluor™ 700	104	BD Biosciences	Cat# 560693; RRID: AB_1727491
Biotin anti-mouse CD4	GK1.5	BioLegend	Cat# 100403; RRID: AB_312688
Biotin anti-mouse CD8a	53-6.7	BioLegend	Cat# 100703; RRID: AB_312742
Biotin anti-mouse/human CD11b	M1/70	BioLegend	Cat# 101203; RRID: AB_312786
Biotin anti-mouse/human CD45R/B220	RA3-6B2	BioLegend	Cat# 103203; RRID: AB_312988
Biotin anti-mouse Ly-6G/Ly-6C (Gr-1)	RB6-8C5	BioLegend	Cat# 108403; RRID: AB_313368
Biotin anti-mouse TER-119/Erythroid Cells	TER-119	BioLegend	Cat# 116203; RRID: AB_313704
Rabbit IgG	N/A	Cell Signaling Technology	Cat# 2729; RRID: AB_1031062
Anti-N6-methyladenosine (m^{6}A) antibody	N/A	Abcam	Cat# ab151230; RRID: AB_2753144
Anti-human CD34 APC/Cyanine7	581	BioLegend	Cat# 343513; RRID: AB_1877169
Anti-mouse APC/Cyanine7 CD14	Sa14-2	BioLegend	Cat# 123318; RRID: AB_10897102
Anti-human CD14 PE/Cyanine7	M5E2	BioLegend	Cat# 982510; RRID:AB_2922652
Anti-human LILRB4	N/A	ABclonal	Cat# A7073; RRID: AB_2767628
Anti-human CD33	2C1	OriGene	Cat# TA506294; RRID:AB_2623704
Anti-human CD45	N/A	Proteintech	Cat# 20103-1-AP; RRID: AB_2716813

Supplementary Table S5
List of main reagents and resources.

Reagent or Resource	Source	Identifier
C57BL/6 mice	GemPharmatech Co. Ltd	Strain NO. N000013
NCG-(Prkdc)KO/KO~(Il2rg)KO/KO mice	GemPharmatech Co. Ltd	Strain NO. T001475
C57BL/6-(Rag1)KO/KO	GemPharmatech Co. Ltd	Strain NO. T004753
C57BL/6-Jab1-flox mice	GemPharmatech Co. Ltd	Strain NO.T005082
B6.Cg-Tg(Mx1-Cre)1Cgn/J mice	From the Laboratory of Haojian Zhang, Wuhan University, Wuhan, China	N/A
C57BL/6-CD45.1-Ptprc mice	From the Laboratory of Haojian Zhang, Wuhan University, Wuhan, China	N/A
C1498	ATCC	TIB-49; RRID:CVCL_3494
MOLM13	DSMZ	ACC-554; RRID: CVCL_2119
MV411	ATCC	CRL-9591; RRID: CVCL_0064
THP1	ATCC	TIB-202; RRID: CVCL_0006
HL-60	ATCC	CCL-240; RRID: CVCL_A794
Kasumi-1	ATCC	CRL-2724; RRID: CVCL_0589
K562	ATCC	CCL-243; RRID:CVCL_0004
Poly(I:C) (HMW)	Invivogen	Cat# TLRL-PIC-5
Actinomycin D	Sigma-Aldrich	Cat# A9415
CSN5i-3	MedChemExpress	Cat# 2375740-98-8
T-5224	MedChemExpress	Cat# 530141-72-1
MLN4924	MedChemExpress	Cat# 905579-51-3
D-Galactose	MedChemExpress	Cat# 59-23-4
D-Luciferin potassium	MeilunBio	Cat# 115144-35-9
Dimethyl sulfoxide	Sigma-Aldrich	Cat# 67-68-5

Hoechst 33342	Sigma-Aldrich	Cat# B2261
7-AAD	BD Biosciences	Cat# 559925
Recombinant Human IL-3	PeproTech	Cat# 200-03
Recombinant Human IL-6	PeproTech	Cat# 200-06
Recombinant Human SCF	PeproTech	Cat# 300-07
Recombinant Human TPO	PeproTech	Cat# 300-18
Recombinant Murine IL-3	PeproTech	Cat# 213-13
Recombinant Murine IL-6	PeproTech	Cat# 216-16
Recombinant Murine SCF	PeproTech	Cat# 250-03
RNA immunoprecipitation Assay Kit	Geneseed	Cat# P0102
SimpleChIP Plus Sonication Chromatin IP Kit	Cell Signaling Technology	Cat# 56383S
Cell Meter Fluorimetric Intracellular Total ROS Activity Assay Kit	AAT Bioquest	Cat# 22903
ROS Assay Kit	Beyotime	Cat# S0033S
Mouse CD117 (cKIT) Positive Selection Kit	StemCell Technologies	Cat# 18757
Bone marrow mononuclear cell isolation kit	Tbdscience	Cat# TBD2013CHU
Phosphate buffer saline	Gibco	Cat# 8115178
Red Blood Cell Lysis Buffer	Solarbio	Cat# R1010
RPMI 1640 medium	Gibco	Cat# 8115178
Penicillin-Streptomycin Solution	Biosharp	Cat# BL505A
MethoCult medium	StemCell Technologies	Cat# M3434
Puromycin	ScienCell	Cat# 0543
Annexin V-FITC/PI apoptosis kit	MultiSciences	Cat# AP101
CCK-8 Cell Proliferation and Cytotoxicity Assay Kit	Solarbio	Cat# CA1210

Fix & Perm intracellular staining kit	MultiSciences	Cat# GAS006/2
TRIzol reagent	Takara	Cat# 9109
Reverse transcription reagent kit	Takara	Cat# RR037A
QuantiTect SYBR Green PCR kit	CWBIO	Cat# CW0957
Fixative Solution 4% formaldehyde	Biosharp	Cat# BL539A
Mouse Genotyping Kit	Vazyme Biotech	Cat# PD101
Nuclear and cytoplasmic protein extraction kit	Beyotime	Cat# P0027
Color PAGE Gel Rapid Preparation Kit	Enzyme organism	Cat# PG112
BCA Protein concentration Assay kit	Beyotime	Cat# P0010S

Supplementary Figure S1

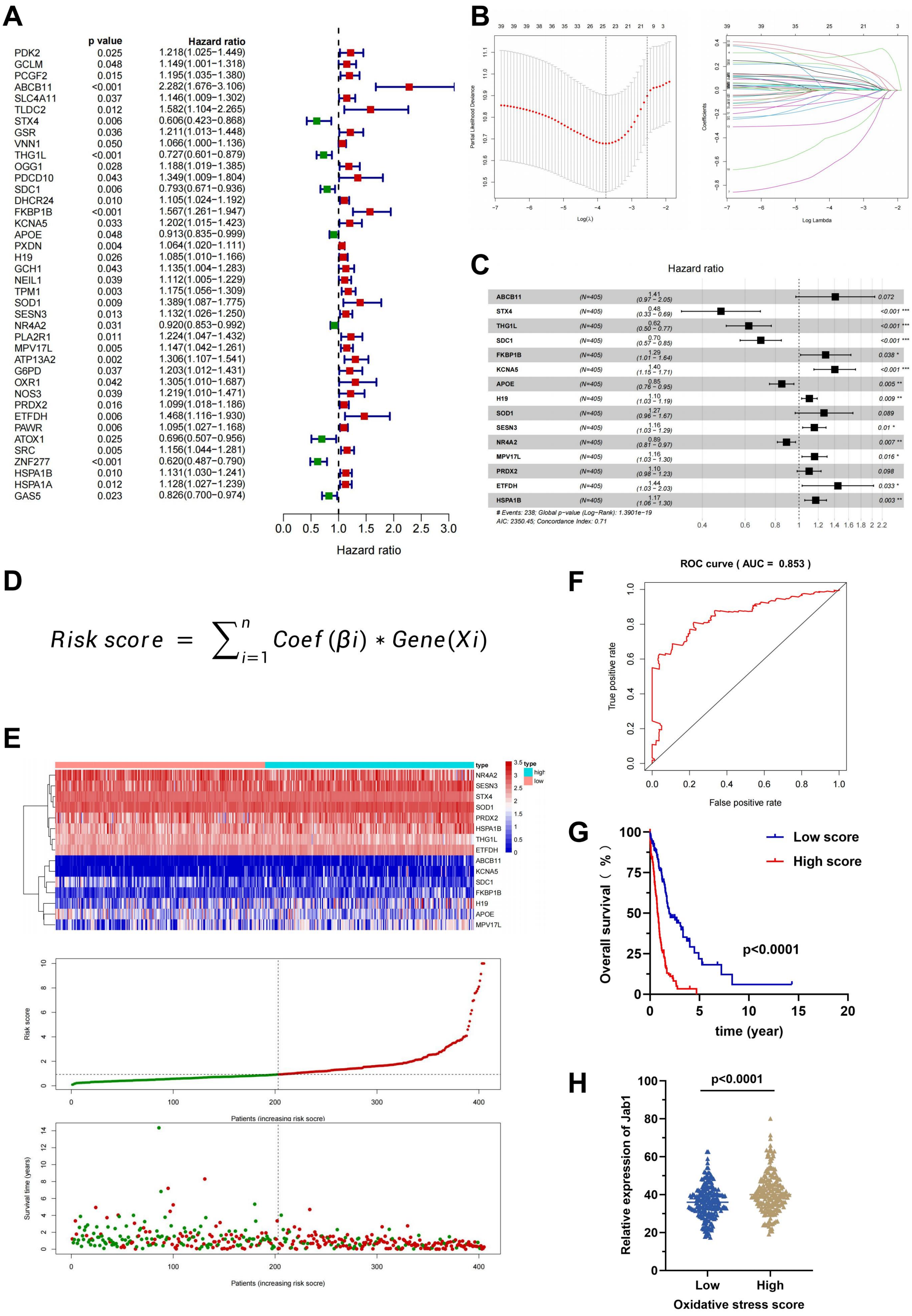

$$Risk\ score\ =\ \sum_{i=1}^{n} Coef\,(\beta i) * Gene(Xi)$$

Supplementary Figure S1. A prognostic model based on oxidative stress-related genes stratifies AML patients and links Jab1 to high oxidative stress.

(A) Univariate Cox regression analysis in the OHSU-AML cohort identifies 40 oxidative stress-related genes significantly associated with overall survival, with 32 genes predicting poor prognosis (HR > 1, red) and others linked to favorable outcomes (HR < 1, green).

(B) LASSO regression analysis determines the optimal penalization parameter (λ) and refines the gene set to the most predictive features.

(C) Multivariate Cox regression confirms 15 key genes as independent prognostic factors incorporated into the risk model.

(D) Risk score calculation formula integrating gene expression and regression coefficients.

(E) Heatmap displays expression patterns of the 15 genes, with risk score distribution and survival status indicating higher mortality in the high-score group.

(F) ROC analysis yields an AUC of 0.853, indicating strong predictive performance of the model.

(G) Kaplan-Meier survival curves show significantly shorter overall survival in patients with high oxidative stress scores ($p < 0.0001$) (by Kaplan–Meier analysis with log-rank test).

(H) Jab1 expression is markedly elevated in the high oxidative stress score group ($p < 0.0001$), reinforcing its association with oxidative stress-driven AML progression (unpaired 2-tailed Student’s t test).

Supplementary Figure S2

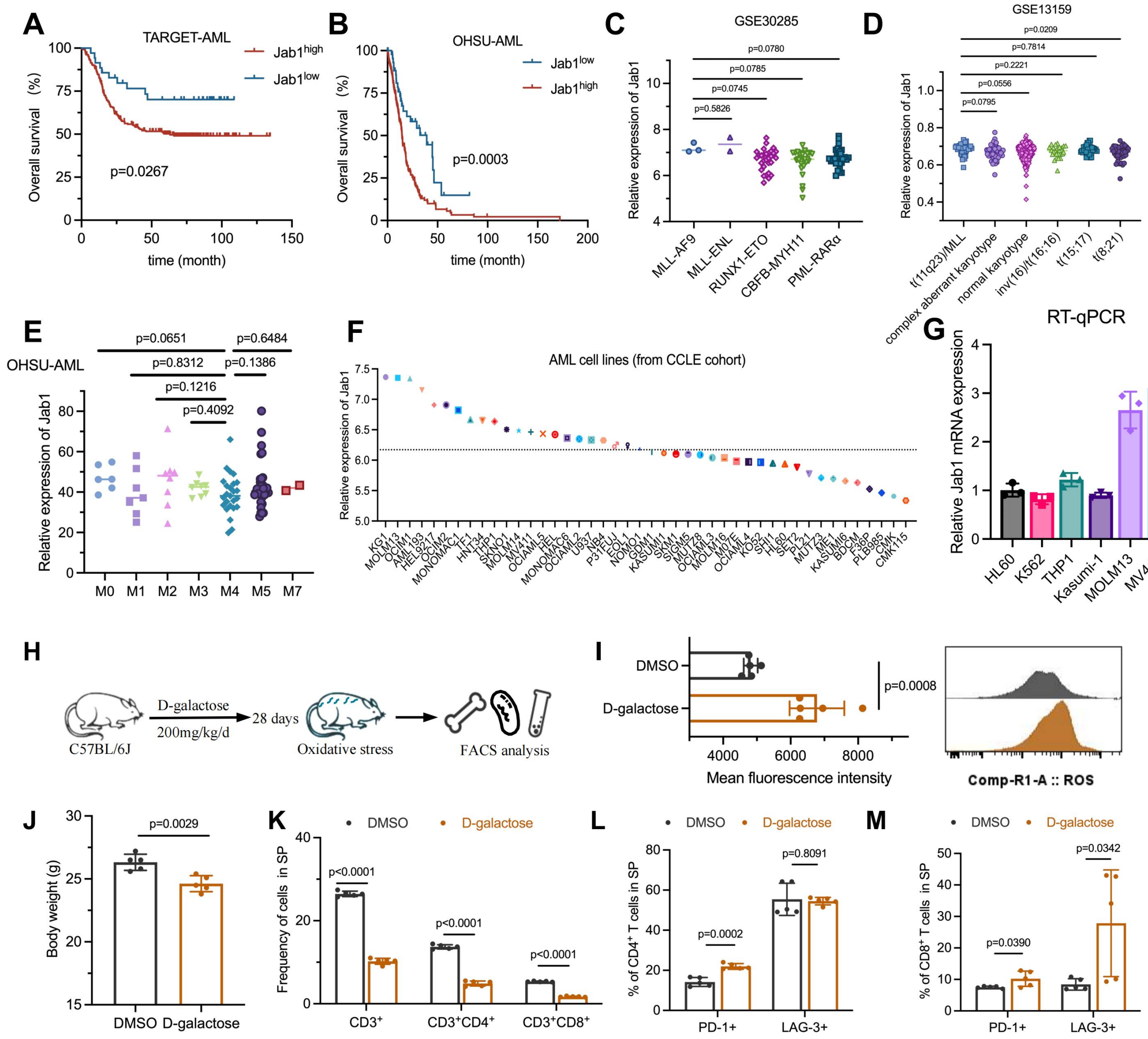

Supplementary Figure S2. Jab1 expression varies across AML subtypes, and D-galactose-induced oxidative stress alters systemic T cell profiles and enhances immunoinhibitory signaling.

(A-B) Kaplan-Meier analysis from the TARGET-AML cohort and OHSU-AML cohort shows that high Jab1 expression is significantly associated with worse overall survival (by Kaplan–Meier analysis with log-rank test).

(C-E) Jab1 expression shows no consistent differences among AML genetic subtypes across the GSE30285, GSE13159, and OHSU-AML cohorts. However, relatively higher Jab1 expression is observed in the FAB M4 subtype (unpaired 2-tailed Student's t test).

(F-G) Jab1 mRNA levels across AML cell lines (from CCLE cohort) and confirmed by RT-qPCR show strong expression in MOLM13 and MV411 cells.

(H) Schematic illustration of the oxidative stress model: C57BL/6J mice were treated with D-galactose (200 mg/kg/day) for 28 days, followed by flow cytometry analysis.

(I) D-galactose treatment significantly increases ROS levels in bone marrow cells, as shown by mean fluorescence intensity ($p = 0.0008$).

(J) Mice treated with D-galactose display reduced body weight compared to controls ($p = 0.0029$) ($n = 5$ per group) (unpaired 2-tailed Student's t test).

(K) Flow cytometry of splenocytes reveals a significant decrease in the frequencies of $CD3^+$, $CD4^+$, and $CD8^+$ T cells in the D-galactose group (all $p < 0.0001$) ($n = 5$ per group) (unpaired 2-tailed Student's t test).

(L) Among $CD4^+$ T cells, the proportion of PD-1^+ cells is significantly elevated after D-galactose treatment ($p = 0.0002$) ($n = 5$ per group), while LAG-3^+ frequency remains unchanged (unpaired 2-tailed Student's t test).

(M) In $CD8^+$ T cells, D-galactose treatment increases both PD-1^+ ($p = 0.0390$) and LAG-3^+ ($p = 0.0342$) populations ($n = 5$ per group) , suggesting enhanced immune exhaustion signatures under oxidative stress (unpaired 2-tailed Student's t test).

Supplementary Figure S3

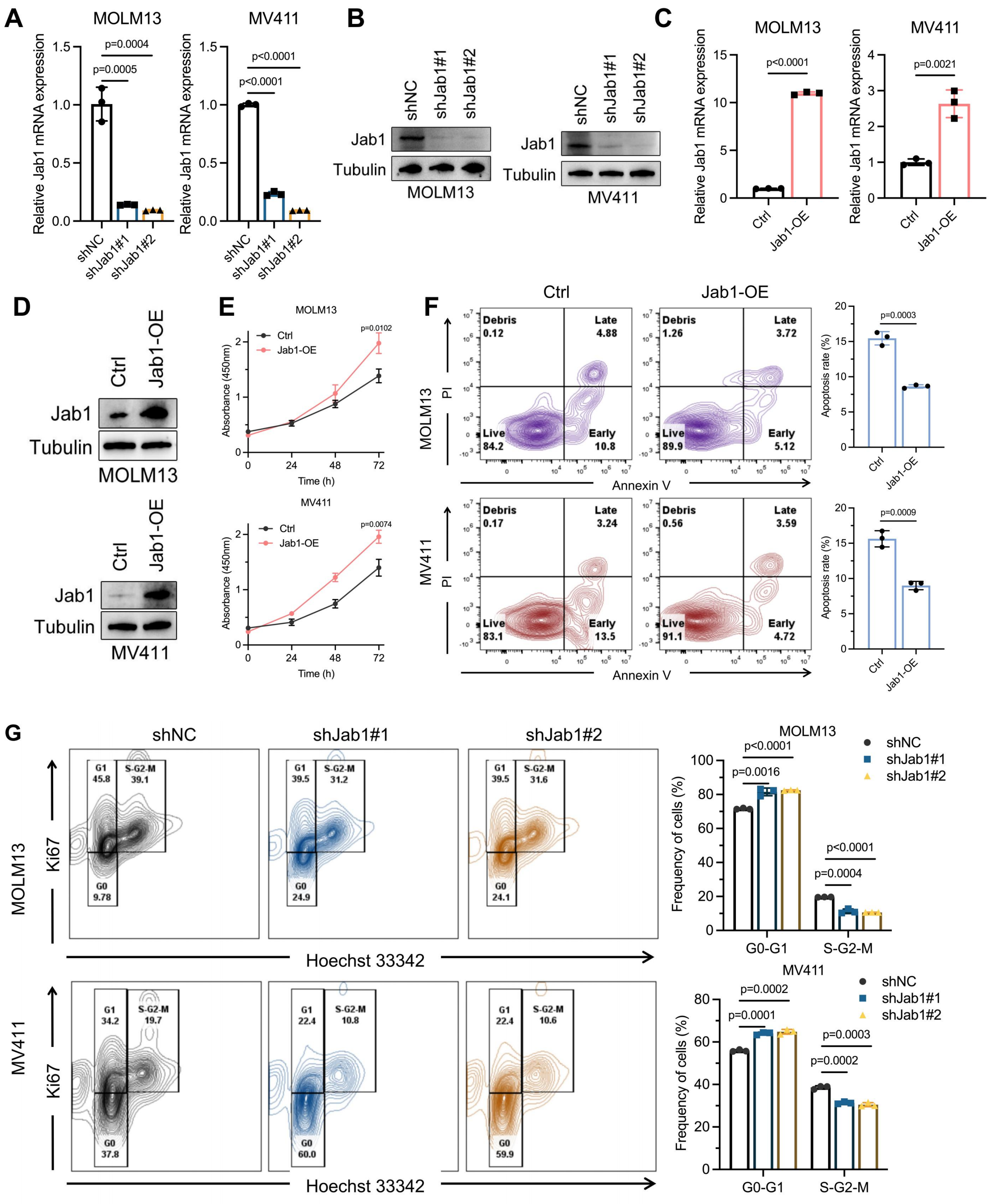

Supplementary Figure S3. Knockdown and overexpression of Jab1 were performed in AML cell lines to investigate their effects on cellular proliferation, apoptosis, and cell cycle distribution.

(A-B) Jab1 knockdown by shRNAs in MOLM13 and MV411 significantly reduces Jab1 mRNA and protein levels.

(C-D) Overexpression of Jab1 (Jab1-OE) increases Jab1 expression in both mRNA and protein.

(E) CCK-8 assays reveal enhanced proliferation upon Jab1 overexpression in AML cells.

(F) Flow cytometry analysis (n = 3 per group) shows that Jab1-OE reduces apoptosis in MOLM13 and MV411 cells (unpaired 2-tailed Student's t test).

(G) Flow cytometry analysis (n = 3 per group) of cell cycle in MOLM13 and MV411 cells upon Jab1 knockdown (unpaired 2-tailed Student's t test).

Supplementary Figure S4

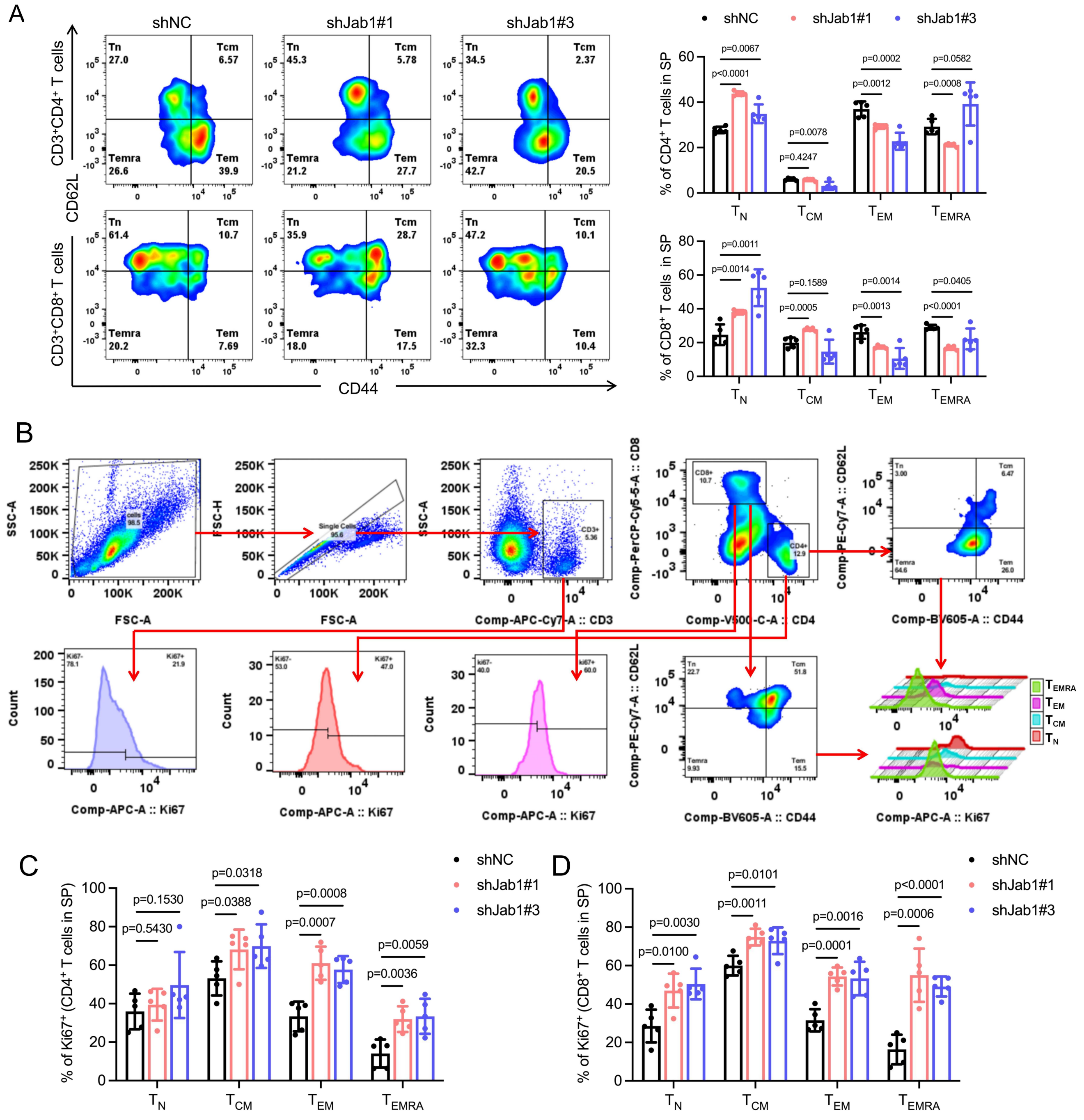

Supplementary Figure S4. Jab1 knockdown alters proliferation and exhaustion marker expression across distinct T cell subsets in AML.

(A) Flow cytometric analysis of T cell differentiation indicates that Jab1 knockdown increases the proportion of naïve T cells (T_N) and decreases effector memory T cells (T_{EM}) in both $CD4^+$ and $CD8^+$ T cell populations, suggesting a shift away from an activated/exhausted phenotype (n = 5 per group, by unpaired 2-tailed Student’s t test).

(B) Flow cytometry gating strategy for identifying $CD4^+$ and $CD8^+$ T cell subsets (T_N, T_{CM}, T_{EM}, T_{EMRA}) and assessing their proliferative status via Ki67.

(C-D) Jab1 silencing significantly increases $Ki67^+$ proportions in $CD4^+$ and $CD8^+$ T cell subsets, particularly in T_{CM}, T_{EM}, and T_{EMRA} populations, indicating enhanced proliferative activity (n = 5 per group, by unpaired 2-tailed Student’s t test).

Supplementary Figure S5

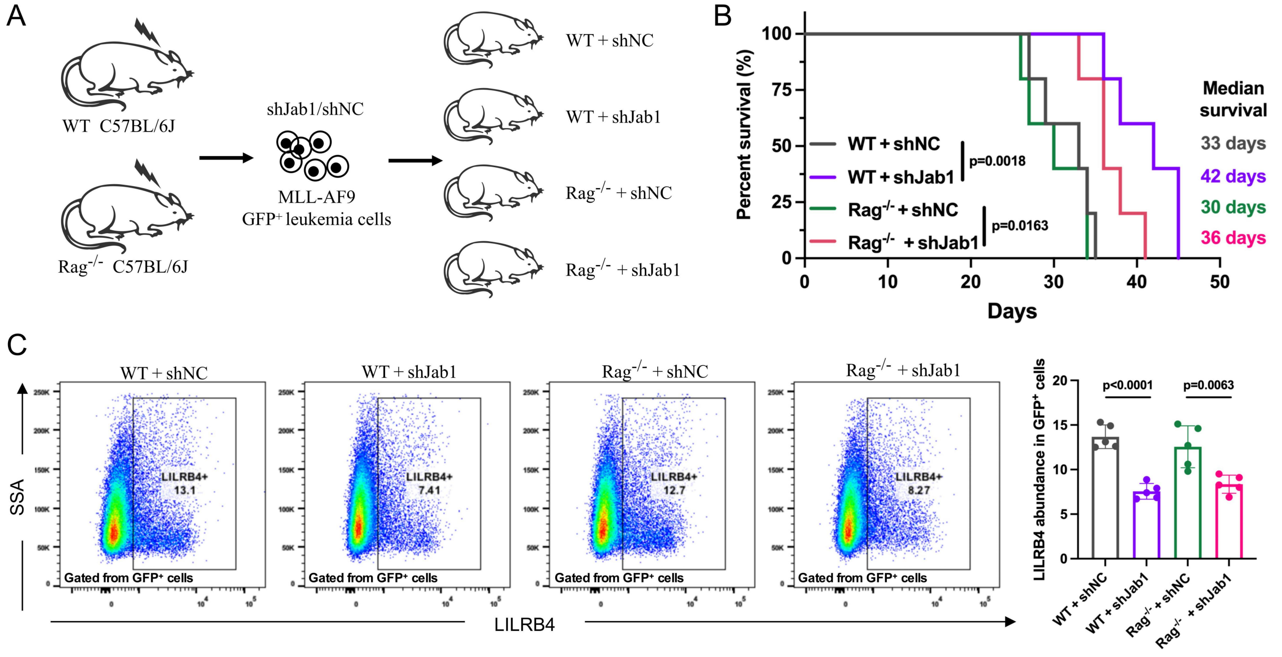

Supplementary Figure S5. Jab1 regulates LILRB4 expression in a cell-intrinsic manner and contributes to AML progression independent of host immunity.

(A) Schematic of the experimental design to evaluate the role of Jab1 in leukemic progression with or without adaptive immunity. MLL-AF9-GFP^{+} leukemia cells transduced with either control shRNA or Jab1-targeting shRNA were transplanted into wild-type (WT) or immunodeficient Rag1$^{-/-}$ C57BL/6J mice.

(B) Kaplan-Meier survival curves show that Jab1 knockdown significantly prolongs survival in both WT and Rag1$^{-/-}$ mice, indicating that the anti-leukemic effect of Jab1 depletion is at least partially cell-intrinsic. Notably, WT mice receiving shJab1-transduced cells exhibited a longer median survival (42 days) than Rag1$^{-/-}$ recipients (36 days), suggesting potential enhancement of immune-mediated control upon Jab1 loss (by Kaplan–Meier analysis with log-rank test).

(C) Flow cytometry analysis of LILRB4 expression in GFP^{+} AML cells from bone marrow reveals that Jab1 knockdown reduces LILRB4 surface levels in both immunocompetent and immunodeficient recipients, supporting a Jab1-mediated, immune-independent regulatory mechanism (n = 5 per group, by unpaired 2-tailed Student's t test).

Supplementary Figure S6

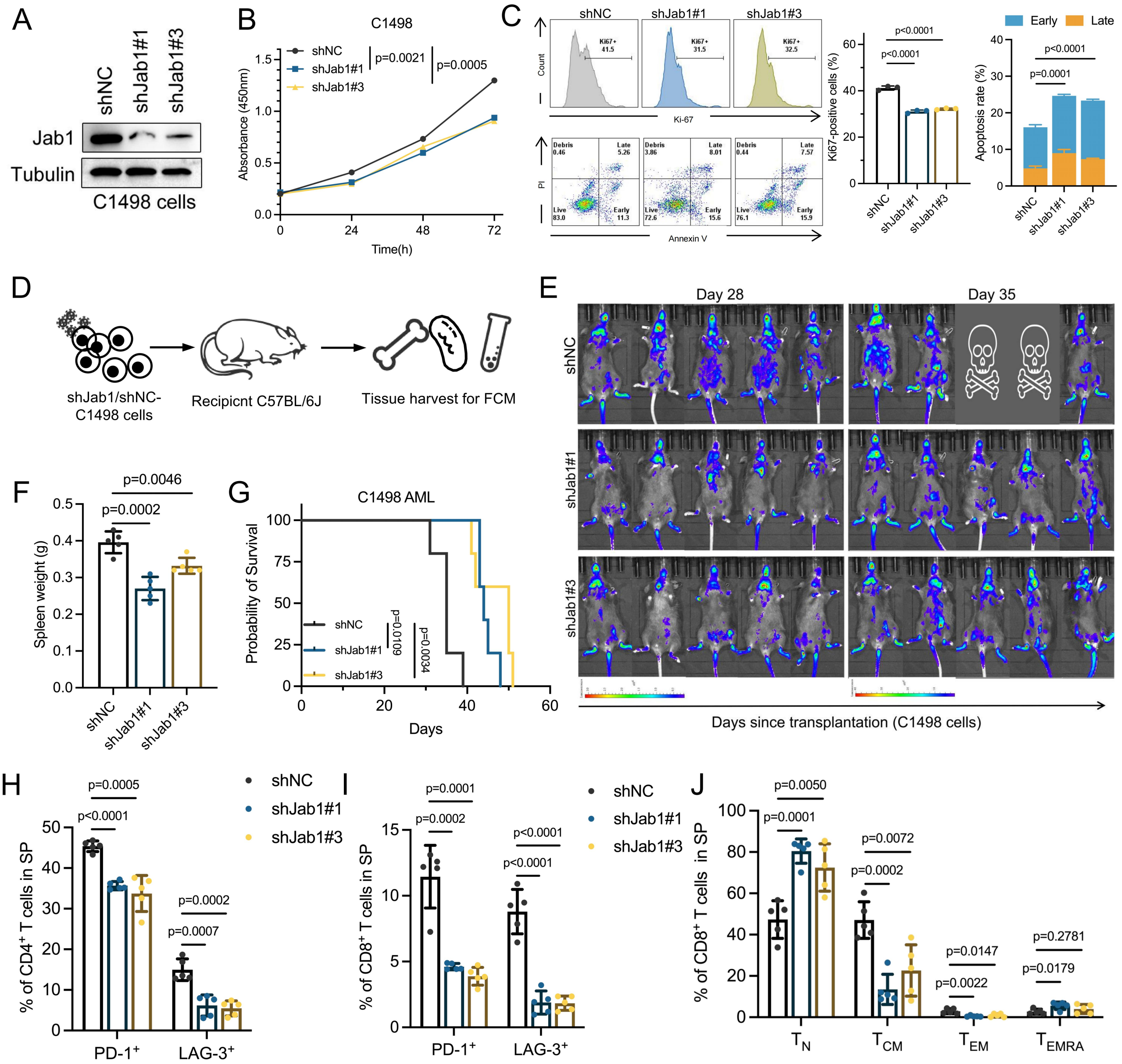

Supplementary Figure S6. Jab1 knockdown suppresses leukemia progression and alleviates T cell exhaustion in a C1498 AML model.

(A) Western blot confirms efficient knockdown of Jab1 in murine C1498 leukemia cells.

(B) CCK-8 assays show that Jab1 knockdown significantly reduces the proliferation of C1498 cells in vitro.

(C) Flow cytometry analysis reveals decreased Ki-67$^+$ proliferating cells and increased Annexin V$^+$ apoptotic cells in Jab1-deficient C1498 cells, indicating impaired cell growth and survival (n = 3 per group, by unpaired 2-tailed Student's t test).

(D) Schematic diagram of the C1498-AML transplant model in C57BL/6J mice using shJab1 or control-transduced cells.

(E) In vivo bioluminescence imaging demonstrates reduced leukemic burden in mice transplanted with Jab1-deficient C1498 cells, particularly at later time points.

(F) Jab1 knockdown significantly reduces spleen weight, reflecting diminished leukemic infiltration (n = 5 per group, by unpaired 2-tailed Student's t test).

(G) Kaplan-Meier survival analysis shows that Jab1 depletion prolongs the survival of mice with C1498-AML (by Kaplan–Meier analysis with log-rank test).

(H-I) Jab1 knockdown leads to a significant reduction in PD-1$^+$ and LAG-3$^+$ exhausted CD8$^+$ T cells in the spleen (n = 5 per group, by unpaired 2-tailed Student's t test).

(J) T cell subset profiling shows an increased frequency of naïve (T_N), and decreased T_{EM} populations, indicating partial restoration of T cell function upon Jab1 silencing (n = 5 per group, by unpaired 2-tailed Student's t test).

Supplementary Figure S7

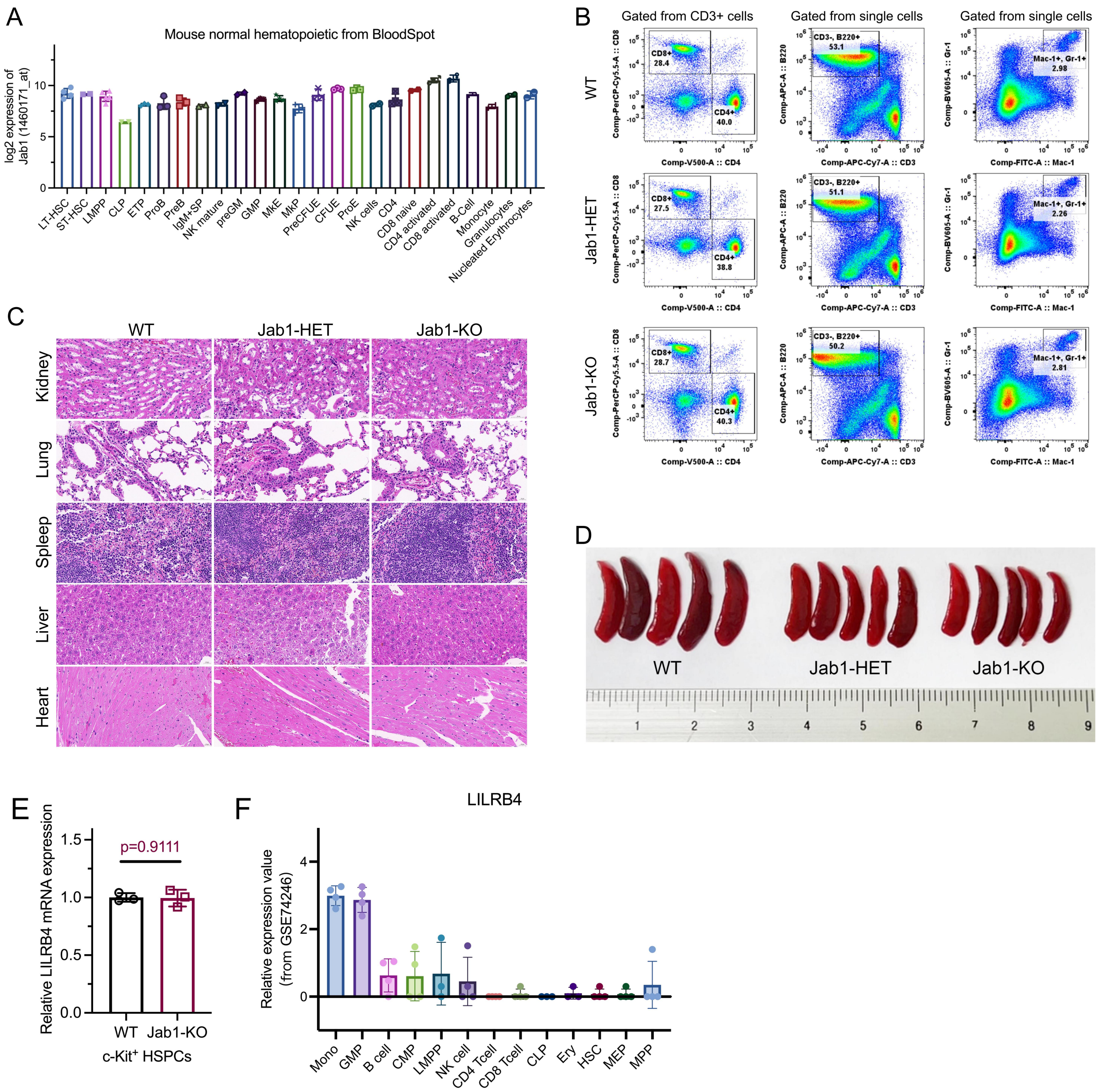

Supplementary Figure S7. Jab1 deficiency does not disrupt normal hematopoietic lineage distribution or tissue morphology.

(A) Public dataset analysis (BloodSpot) shows that Jab1 is broadly expressed across murine hematopoietic stem and mature cell populations.

(B) Flow cytometry analysis of peripheral blood reveals no major alterations in the proportions of $CD4^+$ T cells, $CD8^+$ T cells, B cells, and myeloid cells between WT, Jab1 heterozygous (HET), and knockout (KO) mice.

(C) Histological examination (H&E staining) of major organs—including kidney, lung, spleen, liver, and heart—shows no overt pathological changes in Jab1-HET or Jab1-KO mice compared to WT.

(D) Gross morphology of spleens appears similar across WT, Jab1-HET, and Jab1-KO groups, indicating no evidence of splenomegaly or atrophy.

(E) Quantitative PCR shows that LILRB4 mRNA expression in c-Kit^+ hematopoietic stem/progenitor cells is unaffected by Jab1 knockout (n = 3 per group, by unpaired 2-tailed Student's t test).

(F) Analysis of the GSE74246 dataset confirms that LILRB4 is predominantly expressed in monocytes and GMPs, with minimal expression in early progenitors and lymphoid cells.

Supplementary Figure S8

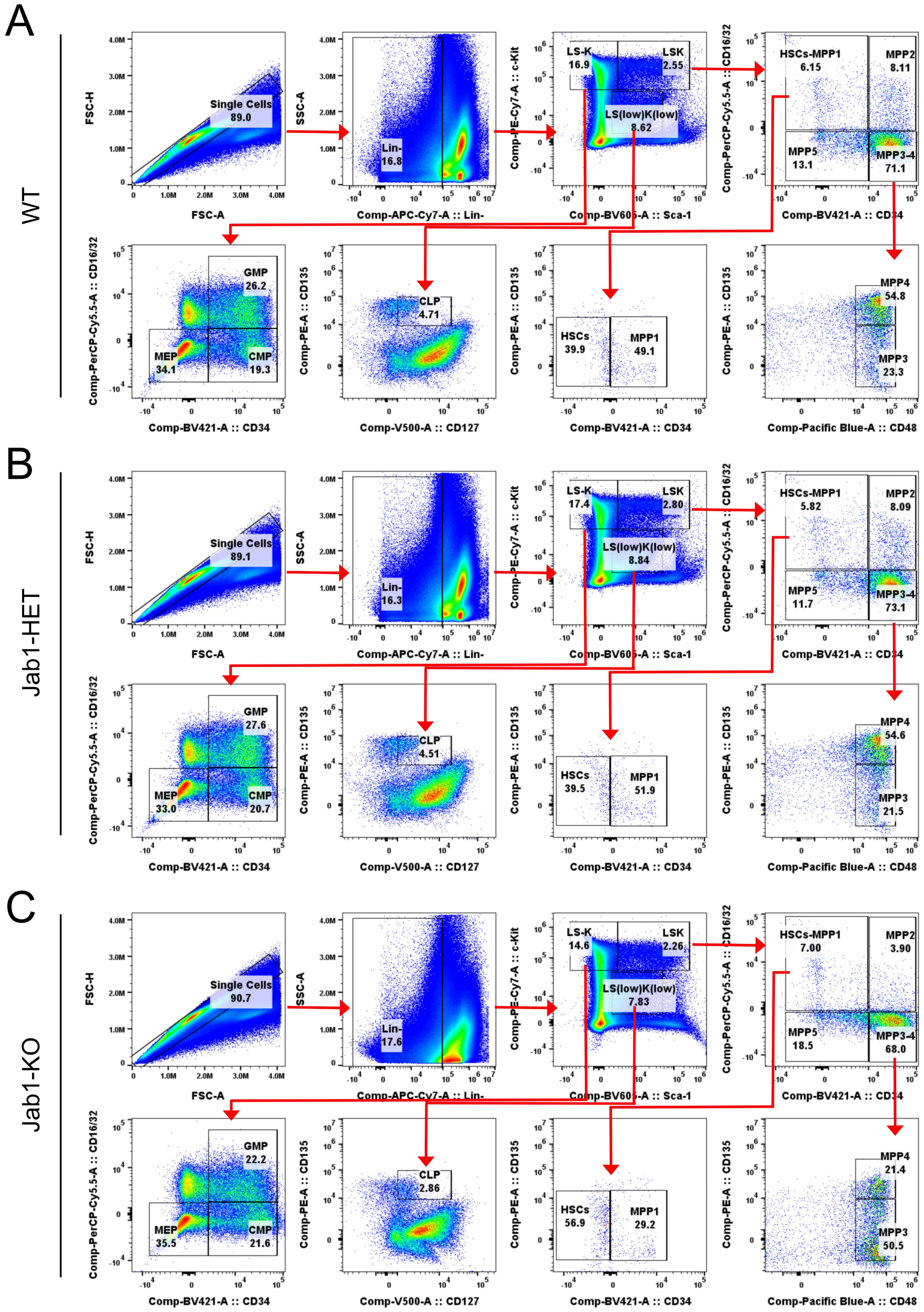

Supplementary Figure S8. Flow cytometric strategy for profiling hematopoietic stem and progenitor cell subsets in bone marrow.

(A) WT mice.

(B) Jab1-HET mice.

(C) Jab1-KO mice.

Supplementary Figure S9

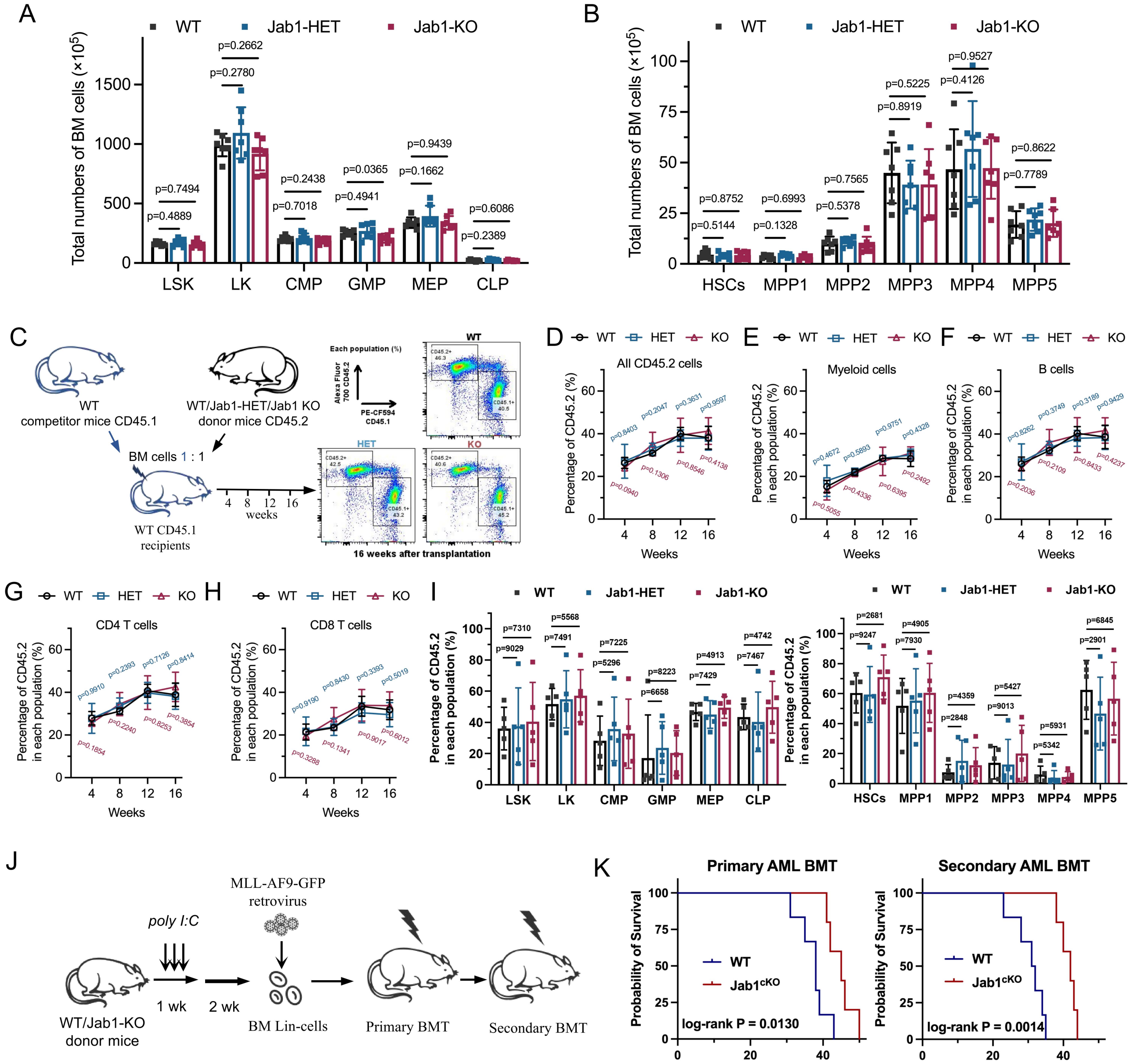

Supplementary Figure S9. Jab1 deficiency does not impair steady-state hematopoiesis or long-term repopulating capacity.

(A-B) Quantification of bone marrow hematopoietic stem and progenitor cell populations in WT, Jab1-HET, and Jab1-KO mice shows no significant differences in most subsets, indicating preserved steady-state hematopoiesis (n ≥ 6 per group, by unpaired 2-tailed Student's t test).

(C) Schematic of competitive bone marrow transplantation: CD45.2$^+$ donor cells from WT, Jab1-HET, or Jab1-KO mice were mixed 1:1 with CD45.1$^+$ WT competitor cells and transplanted into lethally irradiated CD45.1$^+$ recipient mice.

(D-H) Serial flow cytometry of peripheral blood over 16 weeks shows comparable contributions of Jab1-KO donor-derived cells to total leukocytes, myeloid, B cells, CD4$^+$ T cells, and CD8$^+$ T cells (n ≥ 6 per group, by unpaired 2-tailed Student's t test).

(I) Analysis of bone marrow 16 weeks post-transplantation reveals no significant reduction in donor-derived CD45.2$^+$ stem/progenitor populations in Jab1-KO mice, including LSK, CMP, GMP, and MPP subsets, further supporting intact repopulating potential (n ≥ 6 per group, by unpaired 2-tailed Student's t test).

(J–K) In an MLL-AF9-induced AML model, Jab1 knockout delays disease progression and prolongs survival in both primary and secondary transplant recipients, suggesting a functional role for Jab1 in leukemogenesis rather than normal hematopoiesis (Kaplan–Meier analysis with log-rank test) (by Kaplan–Meier analysis with log-rank test).

Supplementary Figure S10

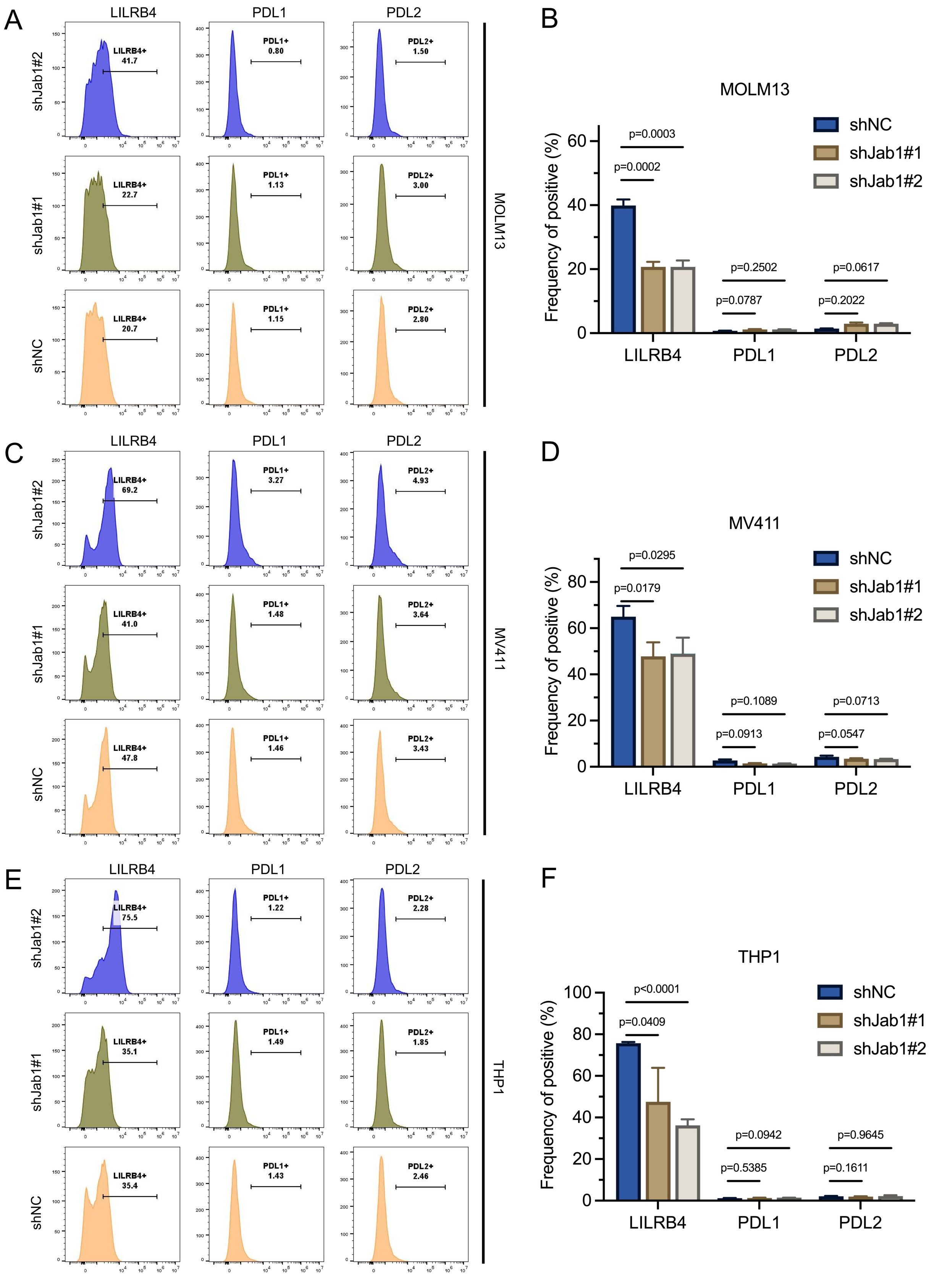

Supplementary Figure S10. Jab1 knockdown selectively reduces LILRB4 surface expression in AML cells.

(A, C, E) Representative flow cytometry plots showing surface expression of LILRB4, PD-L1, and PD-L2 in MOLM13, MV411, and THP1 cells transduced with control (shNC) or Jab1-targeting shRNAs (shJab1#1 and shJab1#2).

(B, D, F) Quantification of flow cytometry results reveals that Jab1 knockdown significantly decreases LILRB4^{+} cell frequency across all three AML cell lines, while PD-L1 and PD-L2 levels remain largely unaffected (n = 3 per group, by unpaired 2-tailed Student’s t test).

Supplementary Figure S11

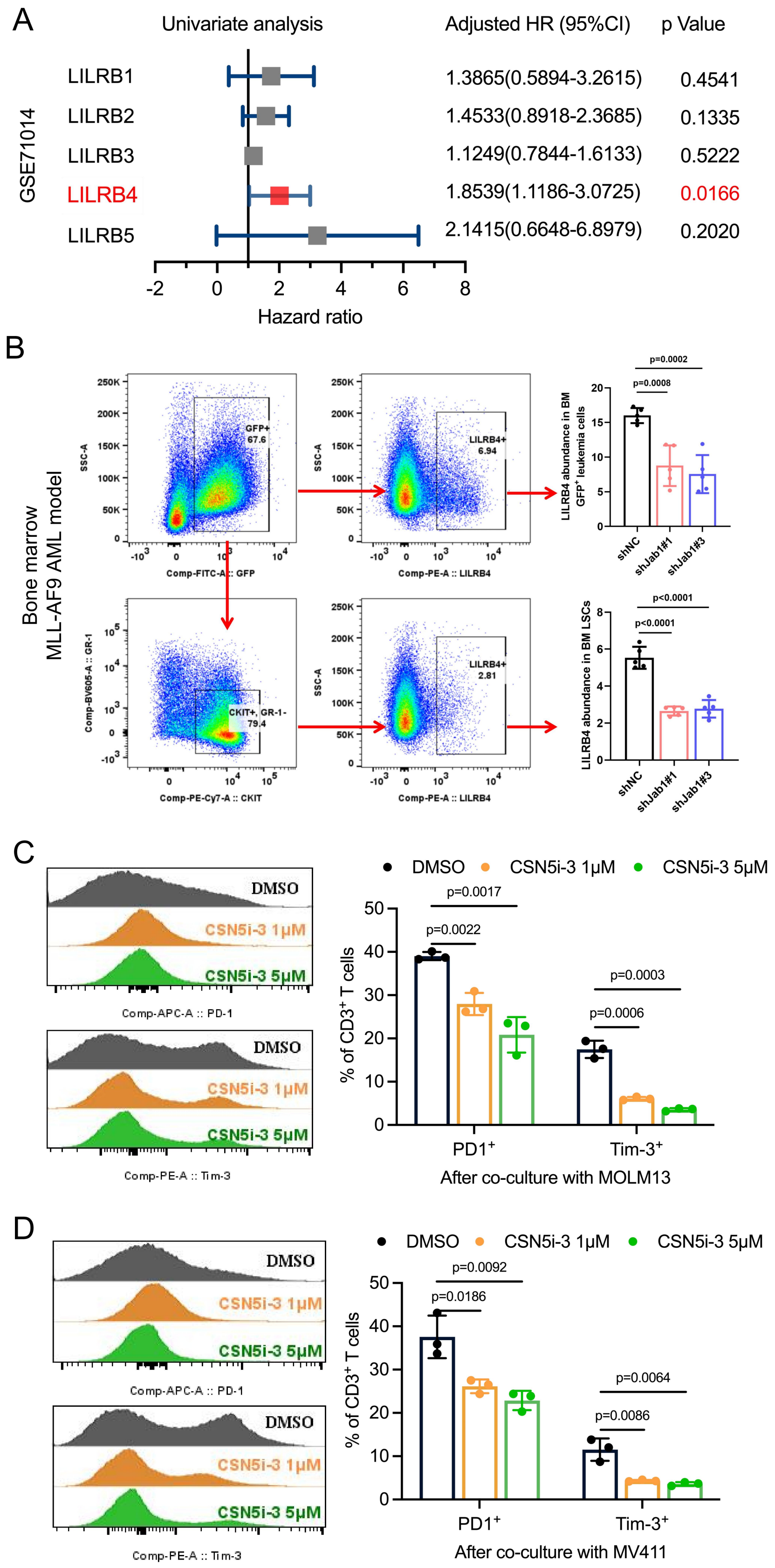

Supplementary Figure S11. Jab1 regulates LILRB4 expression and T cell exhaustion markers in AML

(A) Univariate Cox regression analysis based on the GSE71014 dataset shows that high LILRB4 expression is significantly associated with poor overall survival in AML patients ($p = 0.0166$), while other LILRB family members are not prognostic.

(B) Flow cytometry analysis in MLL-AF9 AML mice reveals that Jab1 knockdown significantly reduces LILRB4 expression in both total GFP^+ leukemia cells and leukemia stem-like cells ($GFP^+Gr\text{-}1^-c\text{-}Kit^+$) from the bone marrow, indicating that Jab1 regulates LILRB4 expression in vivo during leukemogenesis.

(C–D) Flow cytometry reveals reduced expression of exhaustion markers PD-1 and Tim-3 on $CD3^+$ T cells following co-culture with CSN5i-3–treated AML cells ($n = 3$ per group, by unpaired 2-tailed Student's t test).

Supplementary Figure S12

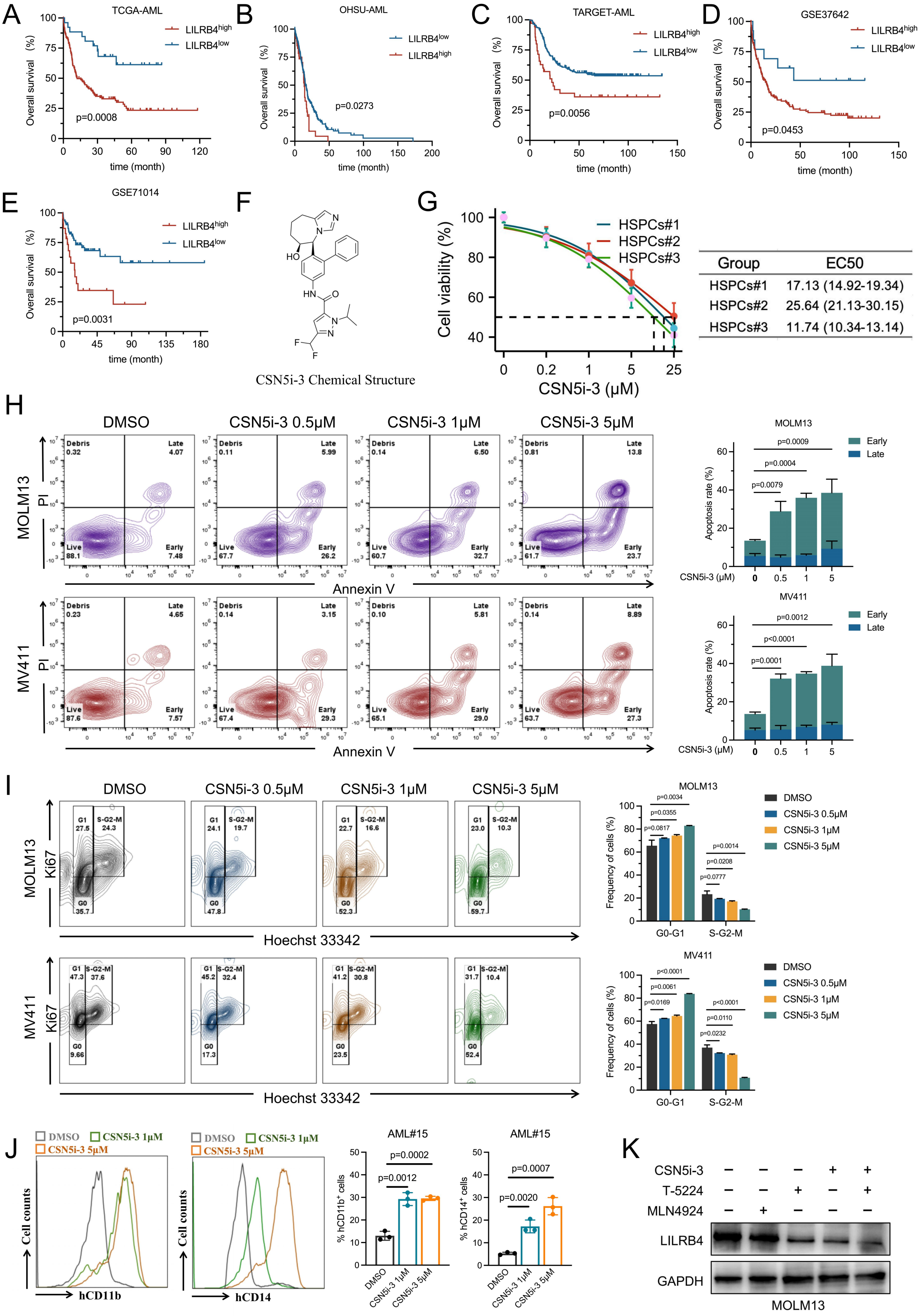

Supplementary Figure S12. Jab1 inhibition by CSN5i-3 impairs AML cell viability and induces apoptosis and cell cycle arrest.

(A-E) Kaplan–Meier survival analyses demonstrate that high LILRB4 expression correlates with worse overall survival across five independent AML cohorts (TCGA-AML, OHSU-AML, TARGET-AML, GSE37642, and GSE71014) (by Kaplan–Meier analysis with log-rank test).

(F) Chemical structure of the Jab1/CSN5 inhibitor CSN5i-3.

(G) CSN5i-3 treatment reduces cell viability in primary human HSPCs in a dose-dependent manner; however, the EC50 values are substantially higher than the effective concentrations required to impair AML cell viability, indicating a therapeutic window between AML targeting and HSPC toxicity.

(H) Annexin V/PI staining shows that CSN5i-3 induces significant apoptosis in MOLM13 and MV411 AML cells in a concentration-dependent fashion (n = 3 per group, by unpaired 2-tailed Student's t test).

(I) Flow cytometry reveals that CSN5i-3 treatment leads to cell cycle arrest at the G0–G1 phase in both AML cell lines (n = 3 per group, by unpaired 2-tailed Student's t test).

(J) CSN5i-3 promotes myeloid differentiation, as indicated by upregulation of CD11b and CD14 surface markers in primary AML blasts (AML#15) (n = 3 per group, by unpaired 2-tailed Student's t test).

(K) Western blot analysis in MOLM13 cells shows that both CSN5i-3 and the AP-1 inhibitor T-5224 reduce LILRB4 expression, while the neddylation inhibitor MLN4924 does not, indicating that Jab1 controls LILRB4 primarily through its transcriptional coactivator function.

Supplementary Figure S13

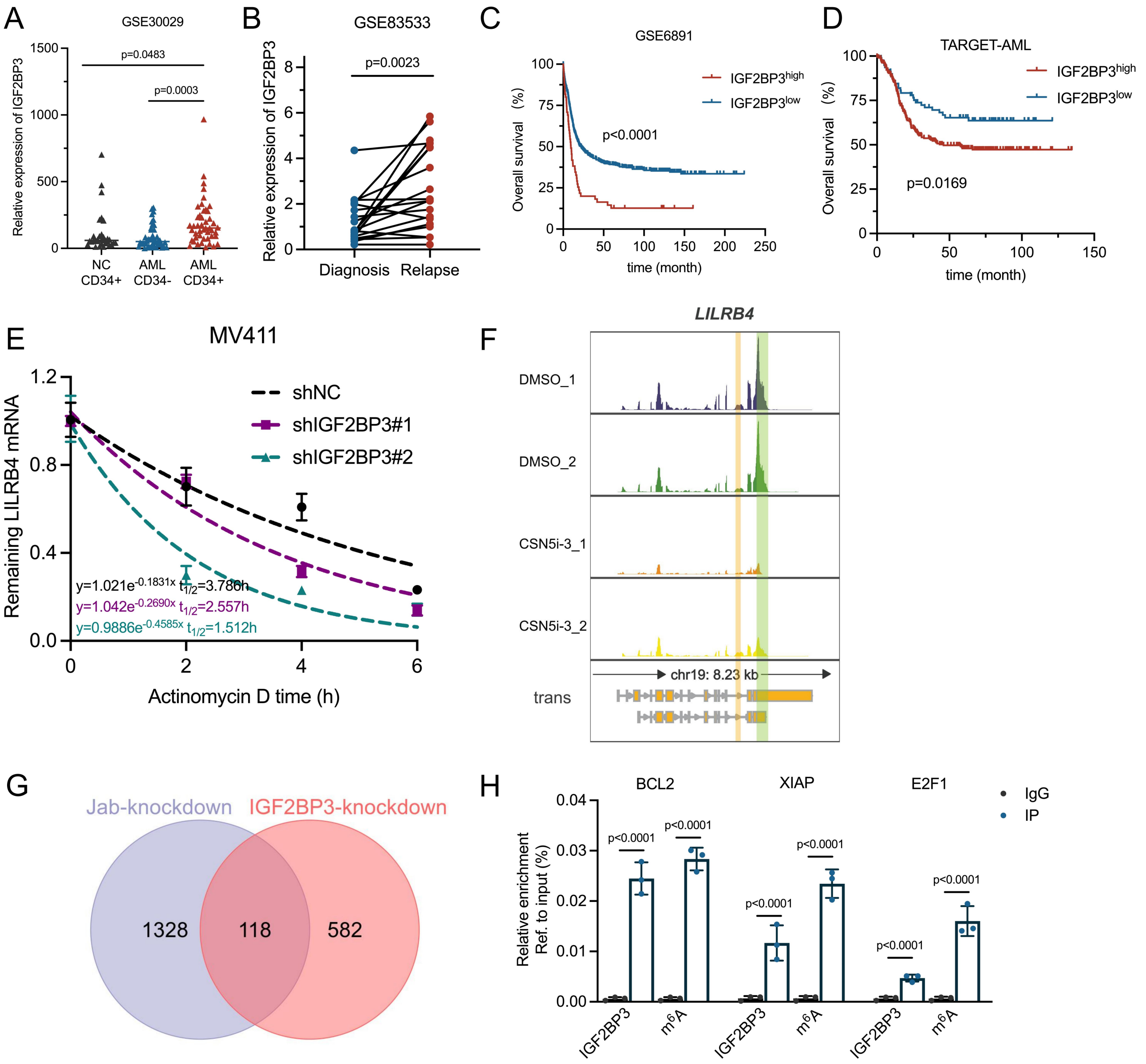

Supplementary Figure S13. IGF2BP3 correlates with poor prognosis and mediates Jab1-driven AML cell survival via LILRB4 stabilization.

(A-B) IGF2BP3 expression is significantly elevated in $CD34^+$ AML cells compared to healthy controls (GSE30092), and further upregulated at relapse in paired AML samples (GSE83533) (by unpaired 2-tailed Student's t test).

(C-D) High IGF2BP3 expression is associated with reduced overall survival in AML cohorts from GSE6891 and TARGET-AML datasets (by Kaplan–Meier analysis with log-rank test).

(E) Actinomycin D transcriptional shutoff assay demonstrates that IGF2BP3 knockdown accelerates LILRB4 mRNA degradation, confirming its role in mRNA stabilization.

(F) IGV tracks reveal loss of m^6A peak enrichment at LILRB4 transcripts in CSN5i-3–treated cells.

(G) Venn diagram shows the overlap between downregulated genes upon Jab1 or IGF2BP3 knockdown, indicating a set of shared downstream targets.

(H) RIP-qPCR analysis confirms that IGF2BP3 binds to m^6A-modified transcripts of BCL2, XIAP, and E2F1—genes known to regulate apoptosis and cell cycle. These findings suggest that beyond LILRB4, the Jab1-IGF2BP3 axis may broadly stabilize oncogenic m^6A-modified mRNAs to promote AML cell survival and proliferation.

Supplementary Figure S14

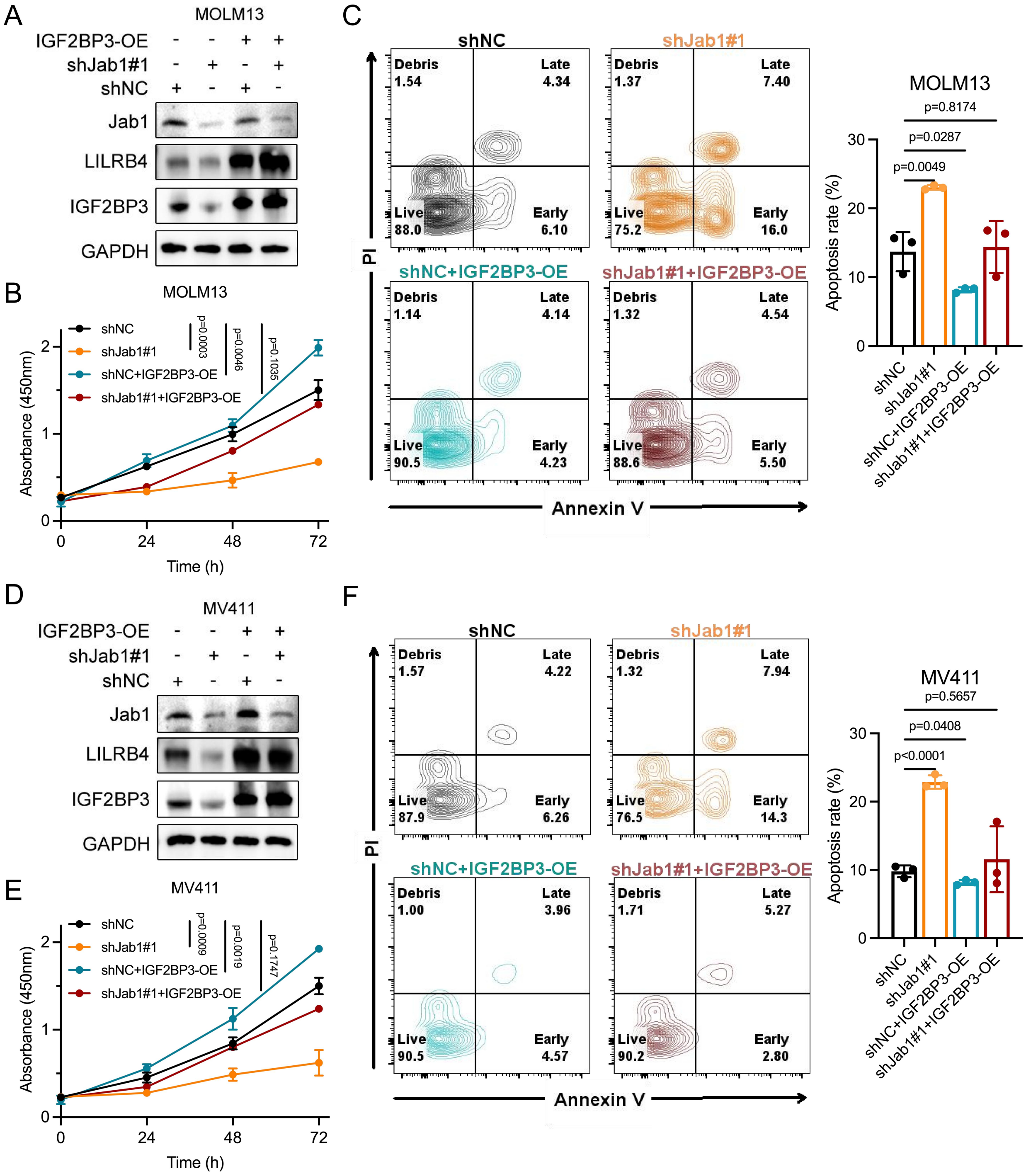

Supplementary Figure S14. Overexpression of IGF2BP3 rescues the growth inhibition of AML caused by Jab1 deficiency

(A) Overexpression of IGF2BP3 restores LILRB4 expression in Jab1-deficient MOLM13 cells.

(B–C) IGF2BP3 overexpression rescues the impaired cell proliferation and increased apoptosis induced by Jab1 knockdown in MOLM13 cells (n = 3 per group, by unpaired 2-tailed Student’s t test).

(C) Overexpression of IGF2BP3 restores LILRB4 expression in Jab1-deficient MV411 cells.

(D–E) IGF2BP3 overexpression rescues the impaired cell proliferation and increased apoptosis induced by Jab1 knockdown in MV411 cells (n = 3 per group, by unpaired 2-tailed Student’s t test).

Supplementary Figure S15

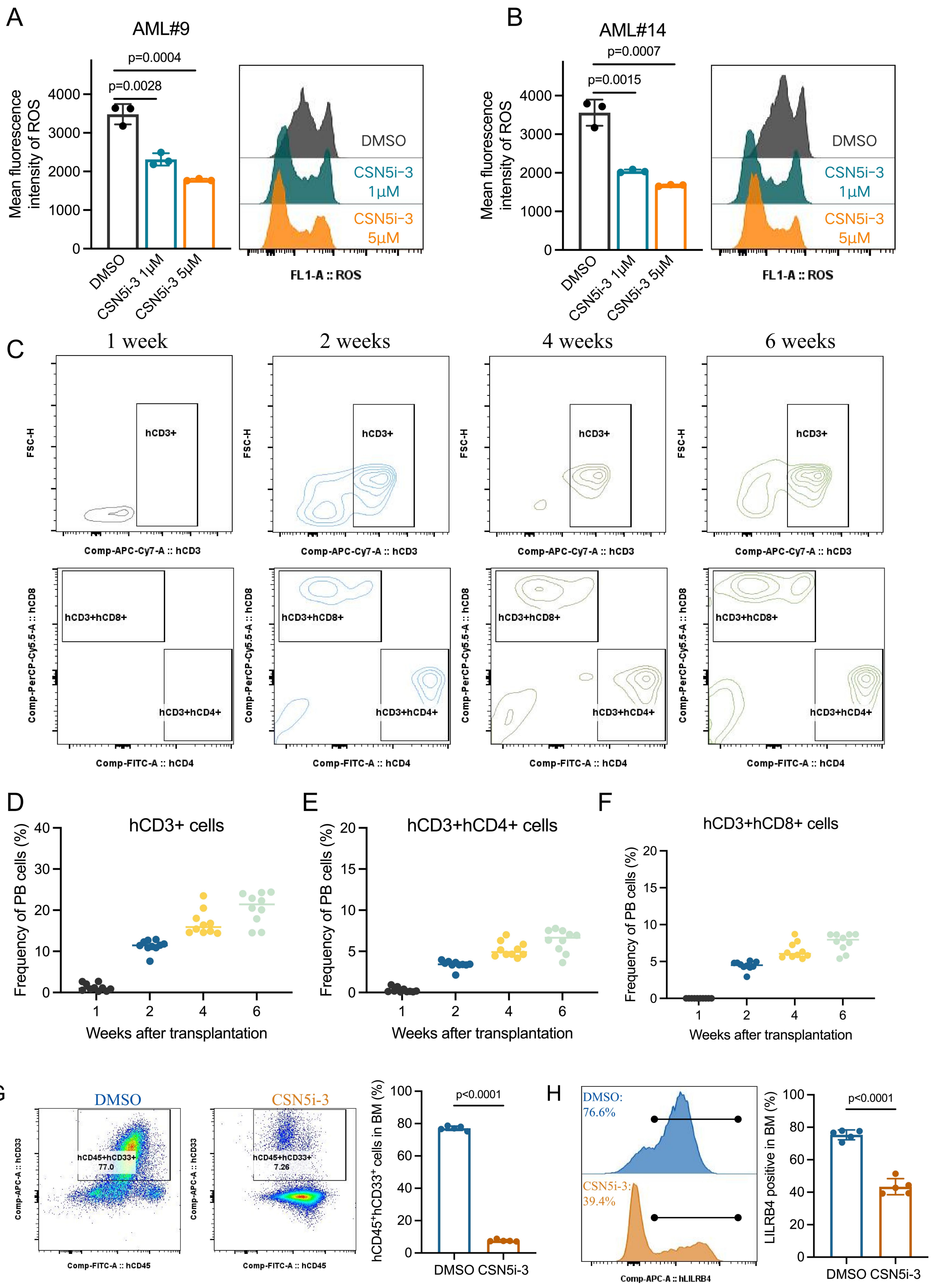

Supplementary Figure S15. CSN5i-3 suppresses ROS in AML cells and impairs human immune cell reconstitution in vivo.

(A-B) CSN5i-3 treatment significantly reduces intracellular ROS levels in primary AML cells (AML#9, AML#14) in a dose-dependent manner.

(C-F) Time-course analysis of human T cell reconstitution in peripheral blood of humanized mice. Flow cytometry reveals progressive emergence of hCD3$^+$, hCD3$^+$hCD4$^+$, and hCD3$^+$hCD8$^+$ T cell populations over 6 weeks post-transplantation.

(G–H) Flow cytometry shows a significant reduction in human CD45$^+$CD33$^+$ leukemic cells and LILRB4$^+$ cells in the bone marrow following CSN5i-3 treatment (n = 5 per group, by unpaired 2-tailed Student's t test).
