# Supplementary material for: Jab1 promotes immune evasion and progression in acute myeloid leukemia models under oxidative stress
Source: J Clin Invest. 2025 Aug 5;135(20):e183761. doi: 10.1172/JCI183761 (PMC12520683; doi:10.1172/JCI183761)

Full unedited blot/gel for Fig. S3B

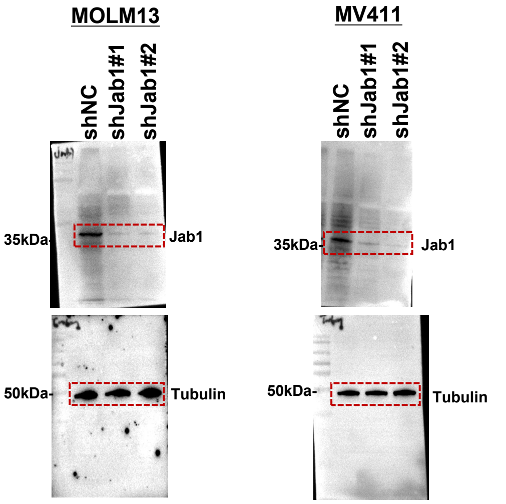

Full unedited blot/gel for Fig. S3D

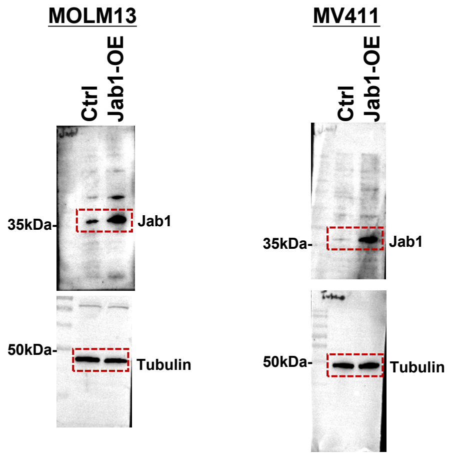

Full unedited blot/gel for Fig. 2B and Fig. S6A

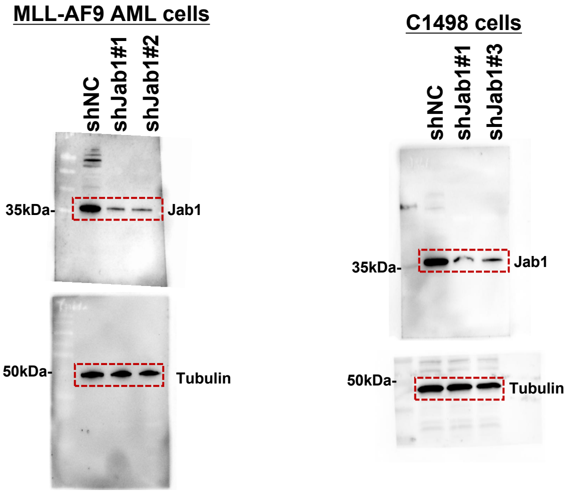

Full unedited blot/gel for Fig. 3A

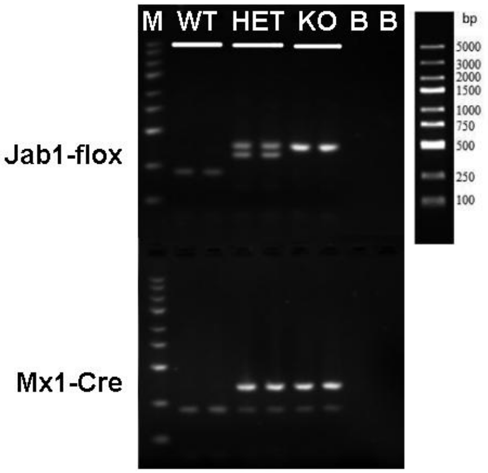

Full unedited blot/gel for Fig. 3A

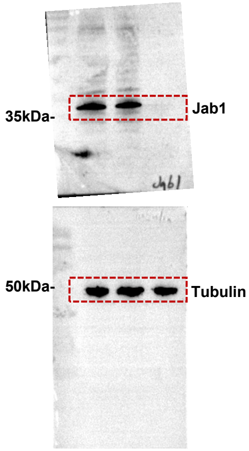

Full unedited blot/gel for Fig. 4H

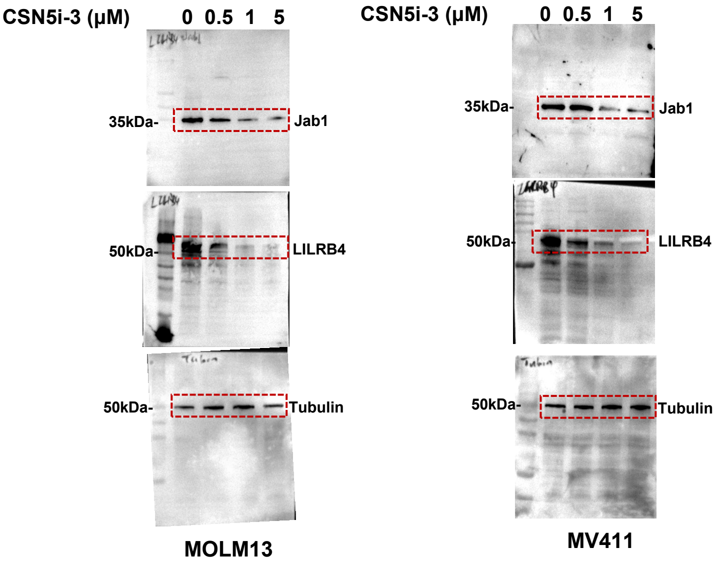

Full unedited blot/gel for Fig. 5F

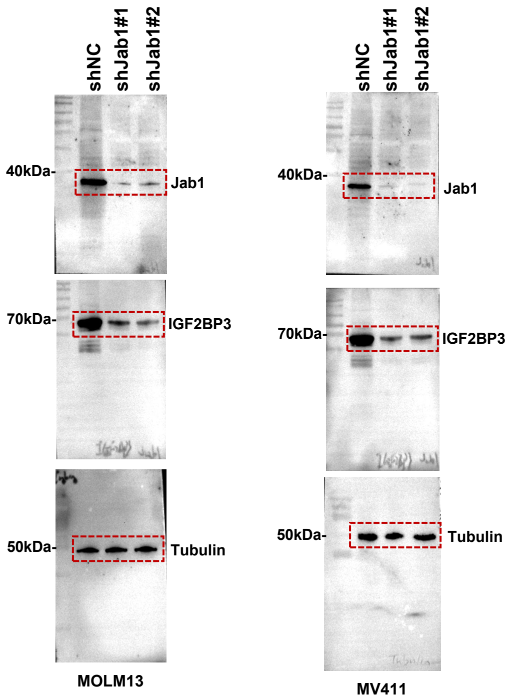

Full unedited blot/gel for Fig. 5F

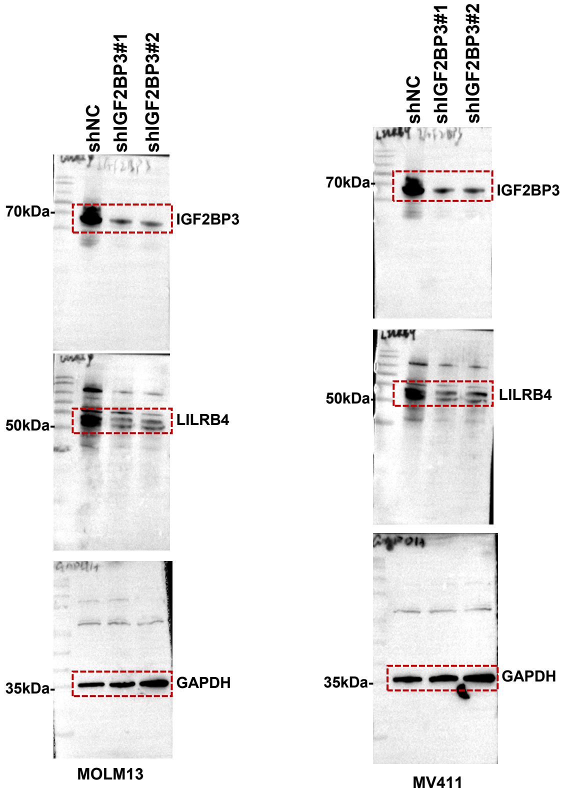

Full unedited blot/gel for Fig. 5K

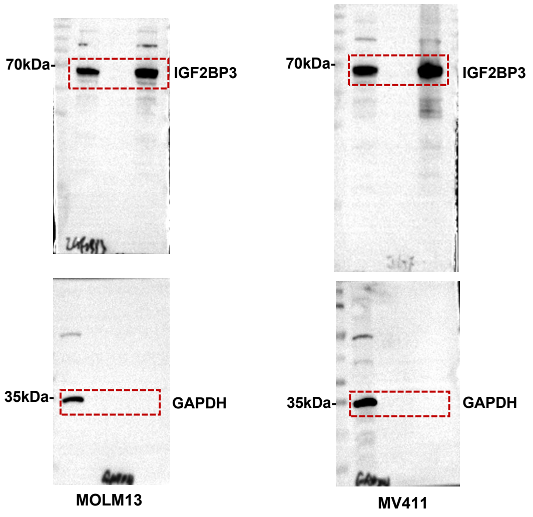

Full unedited blot/gel for Fig. S14A and Fig. S14D

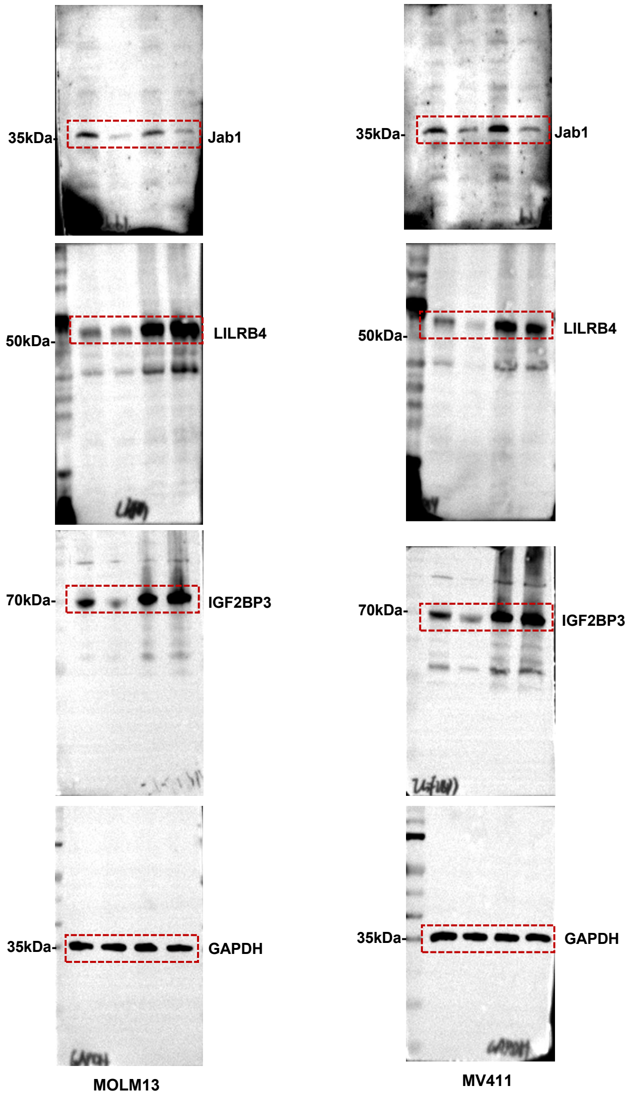

Supplement: Unedited blot and gel images [file jci-135-183761-s020.pdf]
